# Cretaceous bird with dinosaur skull sheds light on avian cranial evolution

**DOI:** 10.1038/s41467-021-24147-z

**Published:** 2021-06-23

**Authors:** Min Wang, Thomas A. Stidham, Zhiheng Li, Xing Xu, Zhonghe Zhou

**Affiliations:** 1 Key Laboratory of Vertebrate Evolution and Human Origins, Institute of Vertebrate Paleontology and Paleoanthropology, Chinese Academy of Sciences, Beijing, China; 2 CAS Center for Excellence in Life and Paleoenvironment, Chinese Academy of Sciences, Beijing, China; 3 University of Chinese Academy of Sciences, Beijing, China

**Keywords:** Evolutionary developmental biology, Palaeontology, Taxonomy

## Abstract

The transformation of the bird skull from an ancestral akinetic, heavy, and toothed dinosaurian morphology to a highly derived, lightweight, edentulous, and kinetic skull is an innovation as significant as powered flight and feathers. Our understanding of evolutionary assembly of the modern form and function of avian cranium has been impeded by the rarity of early bird fossils with well-preserved skulls. Here, we describe a new enantiornithine bird from the Early Cretaceous of China that preserves a nearly complete skull including the palatal elements, exposing the components of cranial kinesis. Our three-dimensional reconstruction of the entire enantiornithine skull demonstrates that this bird has an akinetic skull indicated by the unexpected retention of the plesiomorphic dinosaurian palate and diapsid temporal configurations, capped with a derived avialan rostrum and cranial roof, highlighting the highly modular and mosaic evolution of the avialan skull.

## Abstract (web summary)

In addition to major innovations in their locomotor system, early birds evolved highly derived skulls. Here, Wang et al. three dimensionally reconstruct the skull of a new enantiornithine bird from the Early Cretaceous that illustrates the early avialan transitions in skull morphology and function.

## Introduction

The evolutionary patterns and modes from their first global-scale diversification during the Mesozoic to the >10,000 species of living birds with their great diversity of ecologies, morphologies, and behaviors, are enduring research topics in evolutionary biology^1,2^. While ongoing paleontological discoveries of stem avialans largely have bridged the morphological gap between crown taxa and their closest nonavialan theropod relatives^2^, questions remain surrounding the evolutionary construction and function of the modern avian skull that was assembled heterogeneously and heterochroncially over time through the loss, thinning, and reorganization of various cranial elements and their connections present in the ancestral theropod skull^3–5^. Those evolutionary hypotheses are supported by fossils of stem avialans that exhibit an incompletely known, though vast diversity of cranial morphologies^5–8^. However, uncertainties still remain for key cranial regions, particularly the palatal complex that is functionally integrated in cranial kinesis^9,10^. Avian cranial kinesis, allowing the upper jaw to move independent of the braincase and lower jaw, impacted the radiation of the great diversity of skull shape and ecologies among crown birds^1,9^.

Here, we describe a new enantiornithine bird with an excellent preserved skull from the Early Cretaceous of northeastern China, which demonstrates that the mosaic assembly of the functional aspects of kinesis and complex avian skull osteology must have arisen among crownward lineages.

## Results

IVPP V12707 is interpreted as a late juvenile individual on the basis of its proportionately large skull and orbit, coarse surface texture in the proximal and distal regions of the major limb bones, incomplete fusion of the knee and ankle elements, the partially co-ossified pygostyle, and fused neurocentral sutures on all visible thoracic vertebrae. The degree of bone fusion indicates that IVPP V12707 is more skeletally mature than other recently described juvenile enantiornithines^11–13^.

### Cranial anatomy

**Fig. 1:**
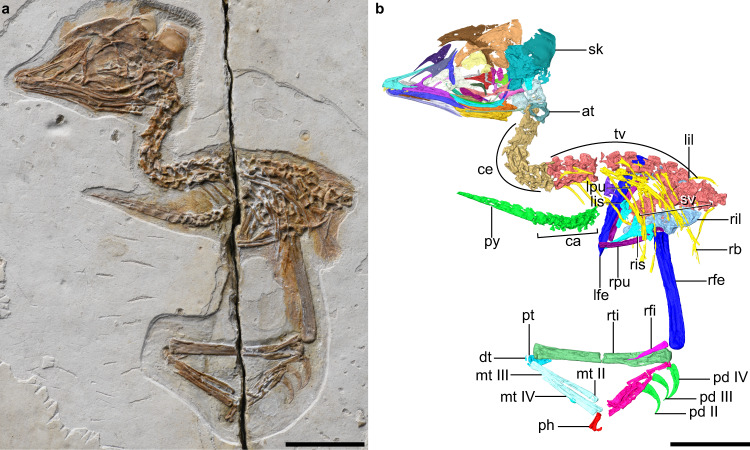
Early Cretaceous enantiornithine IVPP V12707. **a** Photograph. **b** digital reconstruction. at atlas, ca caudal vertebrae, ce cervical vertebrae, dt distal tarsals, lfe left femur, lil left ilium, lis left ischium, lpu left pubis, mt II–IV metatarsals II to IV, pd II–IV pedal digits II to IV, ph proximal phalanx of hallux, pt proximal tarsals, py pygostyle, rb rib, rfe right femur, rfi right fibula, ril right ilium, ris right ischium, rpu right pubis, rti right tibia, sk skull, sv sacral vertebra, tv thoracic vertebrae. Scale bars, 10 mm.

**Fig. 2:**
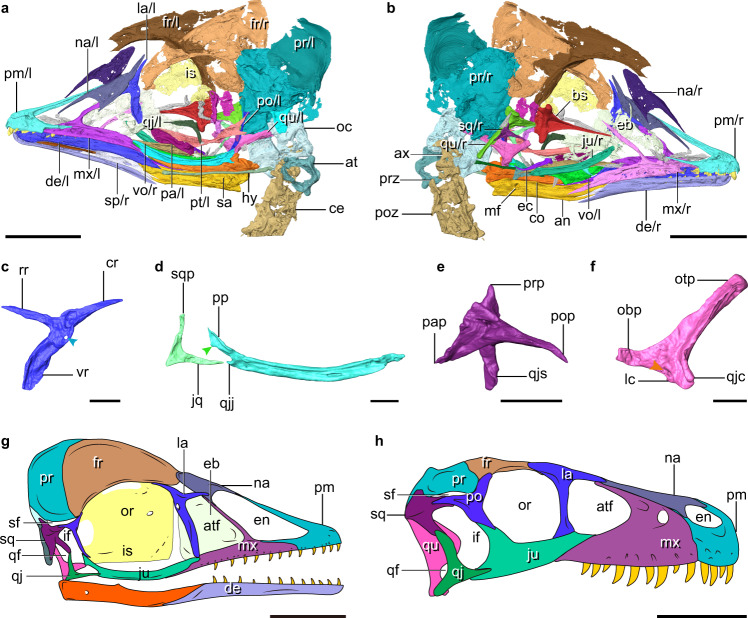
Digital reconstruction of the skull of IVPP V12707. **a, b** Skull and cranial cervicals. **c** Left lacrimal (arrowhead denotes the foramen). **d** Right jugal and quadratojugal (arrowhead denotes the corneal process). **e** Right squamosal. **f** Left quadrate (arrowhead denotes a fossa). **g**, **h** Facial reconstructions of IVPP V12707 (**g**), and *Dromaeosaurus* (**h**; modified from^23^). **c**–**e** lateral view. **f** rostrolateral view. an angular, at atlas, atf antorbital fenestra, ax axis, bs basisphenoid-parasphenoid, ce cervical vertebra, co coronoid process, cr caudal ramus, de dentary, eb ethmoid bone, ec ectopterygoid, en external naris, fr frontal, hy hyoid, if infratemporal fenestra, is interorbital septum, jq jugal process, ju jugal, la lacrimal, lc lateral condyle, mf mandible fenestra, mx maxilla, na nasal, obp orbital process, oc occipital region, or orbit, otp otic process, pa palatine, pap paroccipital process, pm premaxilla, po postorbital, pop postorbital process, poz postzygapophysis, pp postorbital process, pr parietal, prp parietal processes, prz prezygapophysis, pt pterygoid, qf quadrate fenestra, qj quadratojugal, qjc quadratojugal cotyla, qjj quadratojugal process of jugal, qjs quadratojugal process of squamosal, rr rostral ramus, sa surangular, sf supratemporal fenestra, sp splenial, sq squamosal, sqp squamosal process, vr ventral ramus, vo vomer, l/r left/right side. Scale bars, 5 mm (**a**, **b**, **g**), 1 mm (**c**–**f**), 50 mm (**h**).

**Fig. 3:**
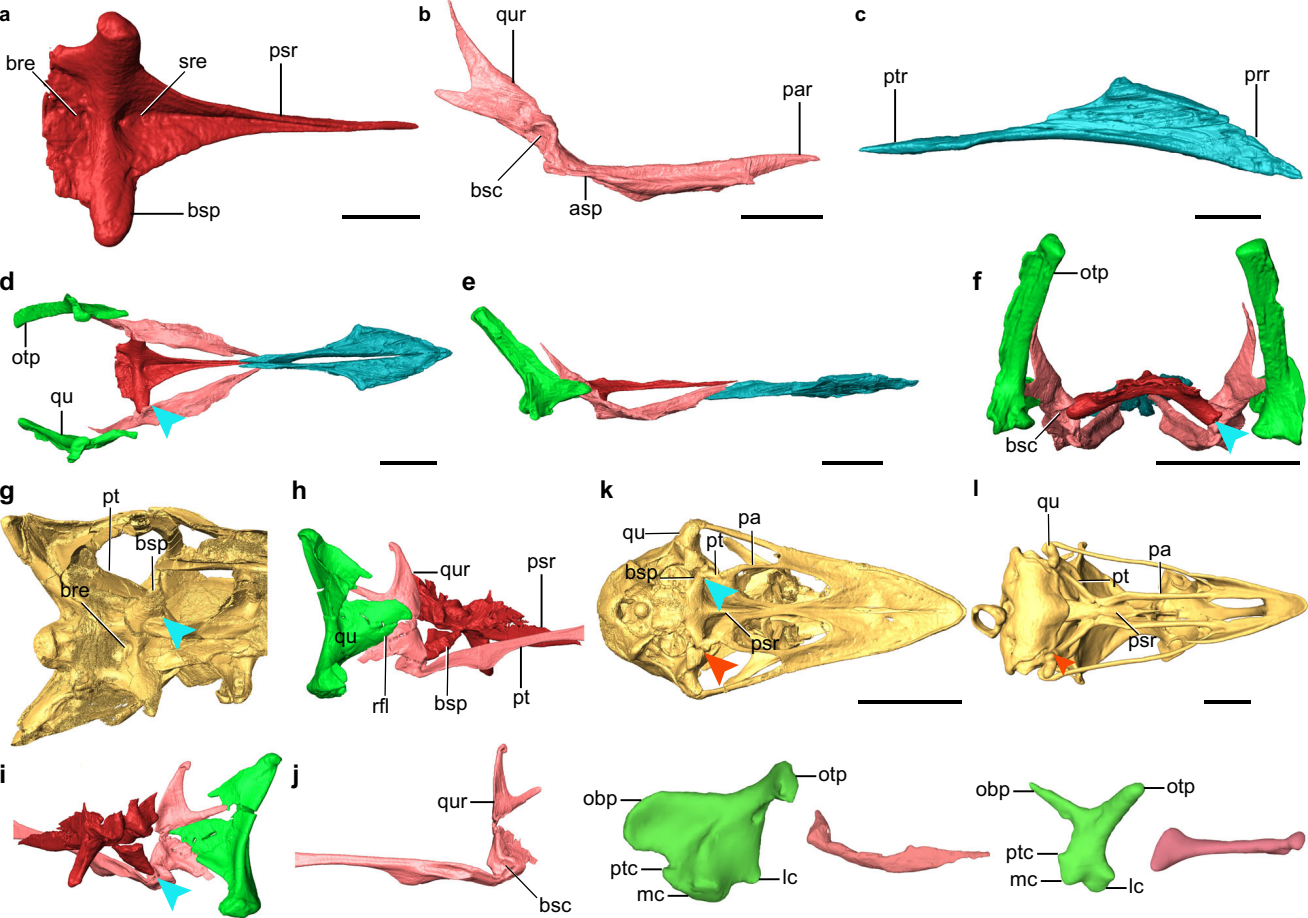
Palatal complex comparison and primitive palate of IVPP V12707. **a**–**f** Digital reconstruction of palatal elements of IVPP V12707: Basisphenoid-parasphenoid (ventral view); **b** Left pterygoid (lateral view); **c** Right vomer (dorsal view); and reconstructed palatal complex in ventral (**d**), lateral (**e**), and caudal view (**f**). **g**–**j** Digital reconstruction of *Linheraptor* (Dromaeosauridae) braincase in ventral view (**h**), palate complex in craniolateral (**h**) and caudomedial view (**i**), and left pterygoid in medial view (**j**). **k**, **l** *Dromaius novaehollandiae* (**k**; Casuariiformes), and *Tragopan caboti* (**l**; Galliformes) in ventral view, with their left quadrate and palatine in lateral view. bre basisphenoid recess, bsc basipterygoid process cotyla, bsp basipterygoid process, mc medial condyle, qur quadrate ramus, par palatine ramus, prr premaxillary ramus, psr parasphenoid rostrum, ptc pterygoid condyle, ptr pterygoid ramus, rfl rostral flange, sre subsellar recess. Scale bars, 5 mm (**a**–**c**), 2 mm, (**d**, **e**), 1 mm (**f**), 50 mm (**k**), 10 mm (**l**). The blue arrows denote the ball-socket articulation between the pterygoid and basisphenoid. The red arrows denote the condylar-based articulation between the quadrate and pterygoid, a derived feature absent in nonavialan dinosaurs and the enantiornithine IVPP V12707.

The premaxillae are partially fused rostrally, but are separated caudally along their frontal processes (Fig. 1a, b and Supplementary Fig. 2a, b). As in *Parabohaiornis*^14^, the maxillary process tapers into an elongate dorsal ramus that overlays a ventral notch and sits in a groove on the lateral surface of the maxilla. As in most other enantiornithines^15^, the facial margin is dominated by the maxilla, rather than the premaxilla as in crown birds^8^. The triradiate maxilla has an elongate dorsal process that contacts the nasal (Supplementary Fig. 2c, d), and together they completely separate the external nares from the antorbital fenestra. The maxilla bears a lateral groove that contains a row of nutrient foramina, ending in a caudoventrally oriented margin that articulates with the jugal. The nasals have well-developed maxillary processes (Supplementary Fig. 2e–g) as in the juvenile enantiornithine LP-4450-IEI from Spain^16^, opposite the condition present in other enantiornithines such as *Falcatakely* and *Longipteryx*^6,15^. The premaxillary process of the nasal extends rostrally to the midpoint of the frontal process of the premaxilla, and together they form the dorsal margin of the external naris. As in *Deinonychus*, *Archaeopteryx* and some enantiornithines^15–18^, the T-shaped lacrimal has rostral and caudal rami that extend rostroventrally and caudodorsally, respectively (Fig. 2c and Supplementary Fig. 3a). The dorsal margin of the lacrimal is notably concave between the two dorsal rami, as in *Pengornis* but unlike other enantiornithines^15^. The robust ventral ramus is convex rostrally and differs from the flat form present in other enantiornithines. The caudodorsal corner of the ventral ramus is perforated by a foramen, which is absent in other known early avialans^15^. The ventral ramus tapers ventrally to a blunt end, lacking the expansion present in LP-4450-IEI and *Falcatakely*^6,16^. The frontals are not fused with each other or to the parietals, and the absence of fusion cannot be attributed exclusively to its ontogenetic stage, because that state is distributed widely among stem avialans^15,19^. The lateral margin of the frontal is thickened and flares laterally, forming the orbital margin. The parietals are vaulted dorsally. The rod-like jugal is bowed ventrally and bears a lateral longitudinal groove (Fig. 2d and Supplementary Fig. 3b–e). In a manner similar to *Archaeopteryx* and non-avialan theropods^20^, the jugal has a robust caudodorsally directed postorbital process, and a quadratojugal process that fails to extend as far caudally as the postorbital process; whereas the quadratojugal process is much longer in some enantiornithines like *Falcatakely*^6^. There is a distinct corneal process on the caudal margin of the postorbital process that is present in *Archaeopteryx* but absent in other enantiornithines^6,20,21^. As in other stem avialans^20^, the L-shaped quadratojugal consists of jugal and squamosal processes that are subequal in length, and it lacks the caudal process present in dromaeosaurids but absent among most other non-avialan theropods^22,23^. The left postorbital preserves only the jugal process which is approximately half of the jugal’s length (Fig. 2a and Supplementary Fig. 3f). The postorbital curves slightly rostrally with a tapering end that articulates with the dorsal margin of the postorbital process of the jugal. That contact demonstrates the presence of an archosaurian plesiomorphy of having the infratemporal fenestra completely separated from the orbit^20,24^. The squamosal, an enigmatic element rarely well-preserved in early avialans, is preserved exquisitely here (Fig. 2e and Supplementary Fig. 4). The bone is morphologically similar to that of nonavialan theropods like *Deinonychus* in having postorbital, quadratojugal, paroccipital, and parietal processes, and the quadratojugal process is inset from the squamosal body as in dromaeosaurids^17,25^. By contrast, the only other known squamosal of an enantiornithine bird (LP-4450-IEI) is triradiate and much more slender^15,16^. The postorbital process tapers rostrally rather than being forked as in *Archaeopteryx* and dromaeosaurids^17,21,25,26^. The quadratojugal process is directed less rostrally than in *Archaeopteryx* and dromaeosaurids, and is excavated caudally by a deep furrow, forming a concavity for the quadrate. As in all enantiornithines^15,27^, the quadrate has a bicondylar mandibular process, and the slender otic process is not divided into the squamosal and otic capitula (Fig. 2f and Supplementary Fig. 5). The orbital process is dorsoventrally broad, lacking the tapering shape characteristic of ornithurine birds^28^. As in nonavialan theropods, but unlike *Ichthyornis*, *Hesperornis*, and crown taxa (Fig. 3)^8,28,29^, a discrete pterygoid condyle is absent, pointing to the lack of articulation between the pterygoid and the mandibular process of the quadrate of the kind in crown birds. That absence of a condylar-based articulation between those bones is demonstrated by the more dorsally positioned ventral edge of the quadrate ramus of the pterygoid relative to the more ventrally placed mandibular condylar region of the quadrate as in *Linheraptor* (Fig. 3), *Deinonychus* and other dinosaurs^10,17^ (see Supplementary Note 1 for additional quadrate description).

The basisphenoid-parasphenoid bears an elongate, rostrally tapering parasphenoid rostrum (Fig. 3a–f). Like some nonavialan theropods^30^, the ventral surface of the parasphenoid rostrum is excavated by a trough along its caudal three-fourths, whereas in crown birds the ventral surface is convex (Fig. 3k, l)^23,31^. As in some nonavialan theropods such as *Velociraptor*^22,23,26,32^—but unlike some dromaeosaurids like *Linheraptor* and crownward avialans (Fig. 3g–l)^25,33,34^, a deep subsellar recess is developed at the base of the parasphenoid rostrum and contains a foramen. The prominent basipterygoid processes project from the caudal end of the rostrum and extend ventrolaterally for a distance beyond the breadth of the basisphenoid body, as in nonavialan theropods (Fig. 3 and Supplementary Fig. 7), paleognaths, and possibly *Gobipteryx*^33,35,36^. By contrast, in *Hesperornis* and most neognaths the basipterygoid processes are quite reduced (Fig. 3l)^29,31^. A large basisphenoid recess lies caudal to a transverse edge connecting the basipterygoid processes, as in *Archaeopteryx* and many nonavialan theropods^18^, and absent in crownward avialans and troodontids^31,32,35^. As in crown birds and dromaeosaurids^28,35^, the dorsolateral surface of the basipterygoid process lacks the basipterygoid recess that is present in *Archaeopteryx* and many nonavialan theropods^18,32^. The pterygoid is nearly identical to that of nonavialan maniraptorans in having a prominent caudodorsally oriented quadrate ramus that extends far dorsal to the palatal surface in a position caudal to the orbit (Fig. 3b, j and Supplementary Fig. 7d, e)^10,17,26,30^. By contrast, the pterygoid is modified into simpler strut-like element in *Hesperornis* and more crownward taxa (Fig. 3k, l)^29,31,33^. An unbifurcated and reduced dorsal process of the pterygoid is preserved in hesperornithiforms^29,37^, and the process of the pterygoid which projects into the medial base of the orbital process of the quadrate in the ostrich and emu (Fig. 3l) may be remnants of this larger quadrate process present in this enantiornithine and nonavialan theropods. The quadrate ramus is caudally bifurcated into a longer dorsal and shorter ventral processes, as in dromaeosaurids such as *Deinonychus* and *Linheraptor* (Fig. 3j and Supplementary Fig. 7d, e)^38^. The cotyla of the pterygoid for articulation with the basipterygoid process is a deep concavity and positioned medially at the base of the quadrate ramus, as in some dromaeosaurids like *Deinonychus*^17^. The lateral margin of the pterygoid bears a notch at a level rostral to the basipterygoid process cotyla that likely represents the point of contact with the ectopterygoid. The rostral part of the pterygoid is twisted 90° relative to the quadrate ramus, forming a leaf-like palatine process with a medially positioned dorsal ridge. Unlike *Gobipteryx*^36,39^, the palatine process is not forked rostrally. The incomplete tongue-shaped left palatine is rotated ventrally around its sutural contact with the pterygoid (Fig. 2a). The paired vomers are not co-ossified along their length (Fig. 3c–f). The rostral parts of the vomers are dorsoventrally compressed and bear diamond-shaped lateral expansions as in some nonavialan theropods like *Tyrannosaurus*^40^, differing greatly from that of other early birds^29^. The caudal halves of the vomers are compressed mediolaterally and taper caudally as they diverge, without the caudodorsal process present in *Sapeornis* and most other nonavialan theropods^7^. A hook-shaped fragment is interpreted as the jugal process of the right ectopterygoid (Fig. 2). The bar connecting the caudalward jugal process and the main body of the ectopterygoid is proportionately much longer than in *Archaeopteryx* and *Falcatakely*^6,7,21^.

The dentary is typical of enantiornithines in having nearly straight dorsal and ventral margins, and a caudoventrally sloping caudal margin (Supplementary Fig. 8a–f)^15^. The triangular splenial is thin and perforated by a foramen at the level of its dorsal apex, as in some enantiornithines (Supplementary Fig. 8g)^27^. The post-dentary mandibular elements are partially fused with faint sutures. Unlike many enantiornithines^15,27^, a coronoid process is developed (Fig. 2a, b and Supplementary Fig. 8h, i). As in juvenile enantiornithines^13^, the surangular is perforated by a foramen, a plesiomorphic feature of nonavialan dinosaurs that is rarely seen among avialans^30^. The rod-like hyoid bones are slightly longer than the post-dentary mandible. The occipital elements appear to be fused with one another.

### Axial skeleton anatomy

The complete presacral vertebral column consists of nine cervical (atlas and axis included) and 11 thoracic vertebrae, comparable with adult enantiornithines^41,42^. The ring-like atlas has short costal processes (Supplementary Fig. 9a, b). The diverging pre- and postzygapophyses form an X-shaped outline in dorsal view. The thoracic vertebrae have completely closed neurocentral sutures and flat intercentral articulations (Supplementary Fig. 9c, d). The rectangular neural spines are dorsoventrally taller than the associated centra. Unlike some enantiornithines^43,44^, the centra are not excavated laterally, which could be related to its ontogenetic stage. The synsacrum consists of seven sacral vertebrae (Supplementary Fig. 9e), intermediate between the counts of juvenile and more mature enantiornithines^12^. The sacral centra are poorly fused, but their transverse processes are connected by lamina. There are nine free caudal vertebrae followed by a pygostyle (Supplementary Fig. 9f–i), a count falling within the range known among enantiornithines^11,13^. The box-like free caudals all retain neural spines that are slightly shorter than their associated centra. The pygostyle consists of eight partially fused vertebrae, and is longer than the combined length of the free caudals, as in the juvenile enantiornithine UFRJ-DG 031 Av^11^. The pygostyle is straight, rod-like, and tapers distally, in a manner similar to other juvenile enantiornithines^11,13,45^. The rostral three vertebrae of the pygostyle exhibit a greater degree of fusion than the more caudal ones. The chevrons are craniocaudally oriented as in *Jeholornis*. Most ribs are preserved in articulation with their corresponding thoracic vertebrae and lack uncinate processes.

### Pelvis and hindlimb anatomy

The ilium is convex cranially and has a preacetabular process that is much longer than the postacetabular process (Supplementary Fig. 10a–c). As in other enantiornithines^27,42^, the ischium has a prominent dorsal process proximally (Supplementary Fig. 10d, e). The pubes are compressed slightly transversely and bowed ventrally (Supplementary Fig. 9f, g). The femur has a shelf-like posterior trochanter diagnostic of the Enantiornithes (Supplementary Fig. 11a, b)^44^. The splint-like fibula terminates far proximal to the midpoint of the tibia. The proximal tarsals are unfused with the tibia (Supplementary Fig. 11c). Metatarsals II–IV are not fused with one another or the distal tarsals (Supplementary Fig. 11d–f). Like other enantiornithines^42^, metatarsal IV is more slender than metatarsals II and III. Digit III is the longest, even surpassing the metatarsals in length. As in other enantiornithines^14,19^, the penultimate phalanges are longer than the proximal ones (pointing to an arboreal lifestyle), and the unguals are strongly recurved.

### Phylogenetic analysis

Our phylogenetic analysis, including all characters which to our knowledge are susceptible to ontogenetic variation were coded as missing for IVPP V12707 (see Methods), places this individual deeply nested within the Enantiornithes (Supplementary Fig. 12).

## Discussion

**Fig. 4:**
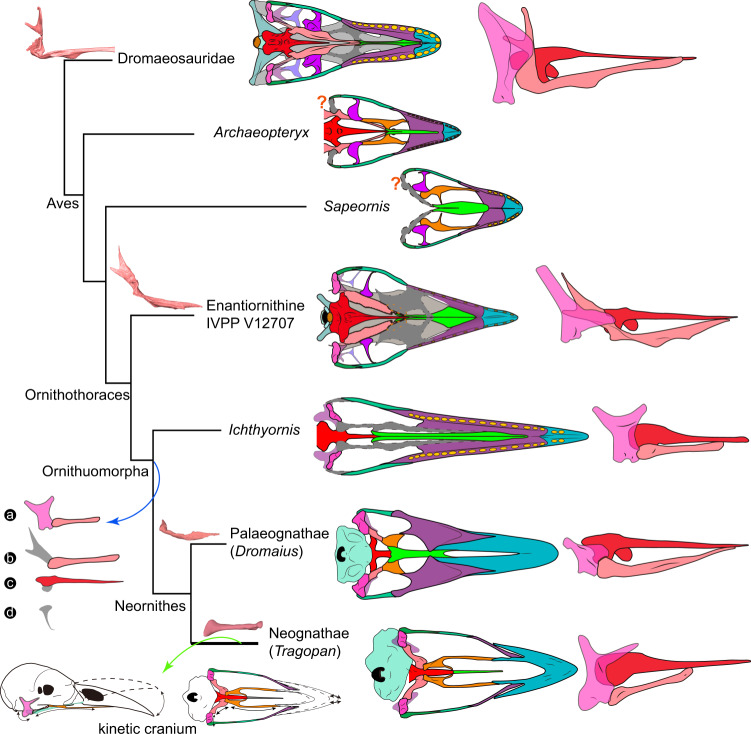
Evolution of avian palatal complex and cranial kinesis. The tree is simplified from the current phylogenetic analysis. The digitally reconstructed pterygoid exhibits almost identical morphology between enantiornithine IVPP V12707 and the dromaeosaurid *Linheraptor*. The modernization of modern avian palatal complex is signified by four stages, which collectively contribute to the avian cranial kinesis: **a** acquisition of the condylar-based contact between the quadrate and pterygoid; **b**–**d** loss/reduction of the quadrate ramus of the pterygoid (**b**), basipterygoid process (**c**), and ectopterygoid (**d**). The available fossils suggest all those modifications took place close to the origin of Ornithuromorpha. Drawings of palatal complex in ventral views except IVPP V12707 and tragopan are modified from literature^7,23,46^ (shaded regions are not preserved, or reduced). The question marks denote the previous reconstructions of a quadrate-pterygoid articulation in *Archaeopteryx* and *Sapeornis* that should be treated with caution.

IVPP V12707 is referrable to the Enantiornithes on basis of preserving following enantiornithine features: the caudal margin of the dentary is caudoventrally sloping; the ischium carries a dorsal process proximally; the femur bears well-developed posterior trochanter; and metatarsal IV is thinner than metatarsals II and III^42,44^. The nearly completely preserved enantiornithine skull provides important information about the cranial morphology and evolution among early birds that has been long hampered by rare and poorly preserved Mesozoic skulls. The extensive postorbital process of the jugal, the squamosal process of the quadratojugal, the large postorbital and squamosal indicate the presence of the plesiomorphic diapsid temporal configuration as in the early diverging pygostylian Confuciusornithiformes and nonavialan dinosaurs (Fig. 2g, h)^20,23,24,35^. The crownward ornithuromorph *Ichthyornis* retains part of the upper temporal bar, but its infratemporal fenestra is confluent with the orbit, approaching the condition in crown birds^8^. Other than those few taxa, the temporal configuration of most early diverging avialans remains poorly known. The morphology of the quadrate has been poorly documented among non-ornithurine avialans, and curiously previous studies (e.g., *Archaeopteryx* and *Sapeornis*^7,21^) have seemingly reconstructed the quadrate with an articulation with the pterygoid near the medial end of the mandibular condyles in a manner as in crown birds, despite the lack of the preservation of those bones or their contacts. By contrast, our three-dimensional reconstruction shows that there is no condylar-based articulation between these two elements given the absence of the pterygoid condyle and the large dorsally extending quadrate ramus of the pterygoid which bypasses the mandibular region of the quadrate, recalling the condition of nonavialan dinosaurs (Figs. 3d–f and 4). The quadrate ramus lies medial to the orbital process of the quadrate with a broad overlap likely forming the scarf joint widely recognized among dinosaurs^10^. Based on this morphology, structure, and relative positioning, we propose that the derived condylar-based pterygoid-quadrate joint utilized in kinesis evolved within crownward ornithuromorphs (Fig. 4).

The palatal complex is functionally vital for avian kinesis in transmitting the force and movement of the cranial and jaw musculature to the upper jaw^9,46^. However, this region is the least understood in transition from nonavialan dinosaur through early birds because of the lack of preservation^3,15^. All of the palatal elements are preserved nearly intact in IVPP V12707, enabling us to reconstruct this region with great fidelity for the first time in early diverging avialans (Figs. 3 and 4); however, the skull roof cannot be reconstructed given that the relevant elements are severely compressed mediolaterally. The morphology of the palatal elements preserved in this enantiornithine demonstrates the retention of a series of plesiomorphic states present in avialan outgroups such as dromaeosaurs (e.g., *Linheraptor*, Fig. 3), troodontids^47^, and oviraptorosaurs^48^. The bony chain of palatal elements from the pterygoid (braced by the basipterygoid process) to the palatine and vomer utilized in cranial kinesis are present and interconnected with each other in this enantiornithine. That palatal kinetic chain lacks the condylar-based contact between the quadrate and pterygoid for rostral-caudal force transmission from the streptostylic quadrate, which is further constrained by the presence of the ectopterygoid. However, the interconnection of most of the palatal elements and likely contact of the orbital process of the quadrate and quadrate ramus of the pterygoid via a scarf joint may point to an origin of the kinetic mechanism along the palate prior to its full establishment along the jugal bar (see Supplementary Note 2 for more discussion).

Kinesis in the skull of extant birds takes many forms as the result of differences in bending zones and skull shapes^9,10,46^. Its evolutionary origin has been clouded by a lack of detailed early records of avialan skulls and perhaps somewhat overzealous researchers interpreting basal birds within the bounds of modern bird structure and function, rather than that of non-avialans. Cranial kinesis with various mechanisms has been proposed for *Archaeopteryx*^49^ and Hesperornithiformes^[29,50^ without a clear evolutionary pattern, and the earliest kinetic cranium probably appeared in the Late Cretaceous ornithuromorph *Ichthyornis*^8^.

The forces required for kinesis in extant birds are transmitted along two pathways through the skull: along the lateral or cheek margin from the quadrate through the jugal-quadratojugal bar to the rostrum^9^; and via a chain of palatal bones from the quadrate to the pterygoid, palatine, and vomer^46^. In this enantiornithine, and basal avialans generally, the continued presence of the diapsid temporal configuration and along with the bracing ectopterygoid contacting the jugal bar would seem to prevent or at least greatly hinder any bending or force transmission from the quadrate along this lateral chain of bones to the rostrum. While that pathway is a known key component of modern kinesis^9^ and clearly absent in this Cretaceous bird, the palatal pathway is important to all forms of avian kinesis^46^. As part of the palatal series of kinetic bones, analysis of the avialan vomer also indicates that early diverging pygostylian *Sapeornis* was not kinetic^7^. Our discovery in this enantiornithine of the absence of the condylar-based contact between the pterygoid and quadrate as required for force transmission and kinesis in living birds leads us to conclude the absence of the kind of kinesis present in crown birds.

The known avialan fossils demonstrate that the kinesis in the avian cranium evolved through modifications signified by the non-synchronous loss of the diapsid temporal configuration and the ectopterygoid (in Ornithuromorpha), reduction/modification of the basipterygoid processes and the quadrate ramus of the pterygoid (outside of Ornithurine), and acquisition of condylar-based contact between the pterygoid and quadrate (outside of Ornithurine), showing that the elements required for cranial kinesis were evolutionarily pieced over a large part of Cretaceous avialan history (Fig. 4). Despite the occurrence of the typical dinosaurian temporal and palatal configurations in this bird^22,26,32^, most other cranial bones of IVPP V12707 display enantiornithine features, including the thin rod-like jugal, the absence of an accessory antorbital fenestra of the maxilla, a quadrate with pointed otic and orbital processes, lightweight jaw bones, and the frontals and parietals that are more dorsally vaulted than in nonavialan theropods (Fig. 4)^15^. The mixture of ancestral and derived cranial traits in a single specimen adds to the tally of instances that mosaicism has greatly shaped the avian skull as a whole^8,51^. It has been shown that the extant bird skull is highly modular where independent regions evolve heterogeneously, with the rostral elements changing the fastest and the palate region (including the pterygoid and quadrate) among the slowest^4,51,52^. The discovery of IVPP V12707 provides substantial further evidence of this mosaic evolutionary hypothesis, along with confirmation of the absence of kinesis among early avialans^7^ by capturing a nonavialan dinosaurian palatal complex surrounded by enantiornithine rostrum, cranial roof, and arboreal skeleton.

The heterogenous evolution among different cranial regions identified in crown birds^51^ may convey those cranial regions with variable developmental versatilities that are ultimately manifested by contrasting amounts of phenotypical disparity under the influence of natural selection^3,51^. The rostrum and its variable kinesis in crown birds exhibits enormous adaptive variations that has allowed them to thrive with diverse ecologies and diets^1^. The long-term evolutionary conservation of aspects of the ancestral dinosaurian skull well into the diversification of birds speaks to a combination of the constraints of developmental pathways, functional necessities, and perhaps an otherwise unrecognized versatility or foundational role of the dinosaurian palate, even when situated within the skull of a small volant vertebrate. Given the success of enantiornithines (as the most diverse Mesozoic avialan clade) and other non-crown avialans through the Cretaceous in habitats around the world^15,53^, these retained evolutionarily primitive morphologies in avialans allowed for a great deal of the ecological, dietary, and phenotypic diversification of avialans up to their demise at the end of the Mesozoic^54^.

The breakdown of constraining non-avian dinosaurian cranium configurations in crown birds has not only conveyed kinesis but also freed the cranial regions to evolve diverse forms that are adapted to diverse habitats^1,3^. Enantiornithines exhibit some taxa with diverse rostra and skull shapes^6,14,15^. However, the plesiomorphic palatal complex and temporal configuration likely offset and constrained much of functional advantages potentially introduced by those disparate rostral morphologies. The mixture of primitive and derived states in this and other enantiornithine skulls may have restricted the clade to less diverse food items and ecologies, but the remodeling of the skulls among Mesozoic ornithuromorphs, along with their more disparate limb proportions and morphologies than those of enantiornithines^53^, could have facilitated their access to additional ecologies by circumventing the constraints imposed by an ancestrally akinetic skull. The interplay among developmental modules and constrains, ecological opportunity, and natural selection may underpin the lineage-specific evolutionary paths of the major lineages of Mesozoic birds. Despite their global conquest and success through the Cretaceous, only the crown group of birds with its derived assemblage of features, allowing for and driving cranial kinesis, survived the end Mesozoic mass extinction, and has thrived ever since.

## Methods

### Locality, and stratigraphic horizon of IVPP V12707

IVPP V12707 is housed at the Institute of Vertebrate Paleontology and Paleoanthropology (IVPP). The specimen was discovered in the Early Cretaceous Jiufotang Formation (120 Ma) near Yixian Country, Jinzhou City, Liaoning Province, northeastern China.

### Computed tomography imaging

In order to increase resolution, we scanned IVPP V12707 three times, focusing on the skull and cranial cervical vertebrae (area I), the dorsal vertebrae and legs (area II), and the tail (area III), respectively. Areas I and II were scanned using the industrial CT scanner Phoenix v-tome-x at the Yinghua Inspection&Testing, in Shanghai, and area III was scanned using the same scanner at the IVPP. Area I was scanned with a beam energy of 180 kV and a flux of 75 μA at a resolution of 7.10 μm per pixel; area II was scanned with beam energy of 190 kV and a flux of 75 μA at a resolution of 10.8 μm; and area III was scanned with beam energy of 110 kV and a flux of 100 μA at a resolution of 10.21 μm. For comparison, we also computed tomography (CT) scanned the skulls of the dromaeosaurid *Linheraptor exquisitus* (holotype, IVPP V12963), and two representatives of crown birds—*Dromaius novaehollandiae* (Palaeognathae: Casuariiformes), and *Tragopan caboti* (Neognathae: Galliformes). *Linheraptor* was scanned using the same machine as IVPP V12707, with beam energy of 240 kV and a flux of 110 μA at a resolution of 29.8 μm per pixel. *Dromaius novaehollandiae* and *Tragopan caboti* were scanned using the micro-computerized-tomography apparatus at the IVPP with beam energy of 380 kV and a flux of 1.5 μA with a resolution of 160 μm per pixel. The resulting scanned data were imported into Avizo (version 9.2.0) for digital segmentation, rendering, and reconstruction, which were then optimized in MeshLab (version 2012.12).

### Phylogenetic analysis

The phylogenetic position of IVPP V12707 was determined using the latest character matrix of the Mesozoic Avian Phylogeny (MAP) project, which is actively being maintained and expanded by the avian evolution research team of the IVPP^53^. Given that IVPP V12707 is an ontogenetically late juvenile, we have cautiously coded any characters likely influenced by ontogenetic variation as missing entry, such as the limb proportions, and the degree of bone fusion. The phylogenetic result present here should be treated cautiously because morphological changes during the ontogeny of stem avialans are not fully understood. The revised matrix consists of 280 morphological characters and 81 terminal taxa, including 78 Mesozoic avialans (Supplementary Notes 3 and 4). The phylogenetic analysis was conducted using the TNT v. 1.5 software package^55^, with all characters equally weighted under parsimony. The “New Technology search” algorithm with sectorial search, ratchet, tree drift and tree fusion using the default settings in TNT was performed, and the minimum tree length was found in ten replicates to recover as many tree islands as possible. Those resulting trees were fed into a second round of branch-swapping using the traditional tree-bisection-reconnection method to find the most parsimonious trees (MPTs). We calculated the Bootstrap and Bremer values as the support indices. The absolute bootstrap values were calculated using 1000 replicates using the same settings as the primary search, and the Bremer values were calculated using the Bremer script in TNT. The phylogenetic analysis produced 1344 MPTs with a length of 1404 (Consistency index = 0.276, Retention index = 0.662). The strict consensus tree is well resolved (Supplementary Fig. 12), and the interrelationships of the major clades are consistent with recent phylogenetic studies^6,8,41^. IVPP V12707 was recovered in a relatively well-nested position within the Enantiornithes, as a sister taxon to *Gretcheniao*.

### Reporting summary

Further information on research design is available in the Nature Research Reporting Summary linked to this article.

## Supplementary information

Supplementary Information

Reporting Summary

## Data Availability

The specimen (IVPP V12707) described in this study is archived and available on request from the Institute of Vertebrate Paleontology and Paleoanthropology (IVPP), Chinese Academy of Sciences, Beijing, China. The three-dimensional models (STL) are archived and available on MorphoBank (project no. 3972), or from the corresponding author. The data matrix used in the phylogenetic analysis is provided in Supplementary Notes 3 and 4.
