# Supplementary material for: Cretaceous bird with dinosaur skull sheds light on avian cranial evolution
Source: Nat Commun. 2021 Jun 23;12:3890. doi: 10.1038/s41467-021-24147-z (PMC8222284; doi:10.1038/s41467-021-24147-z)
Supplement: Supplementary file 1 — Supplementary Information [file 41467_2021_24147_MOESM1_ESM.pdf]

Supplementary Information for:

Cretaceous bird with dinosaur skull sheds light on avian cranial evolution

CONTENTS:

Supplementary Figures

Supplementary Note 1–4

Supplementary Table 1

Supplementary references

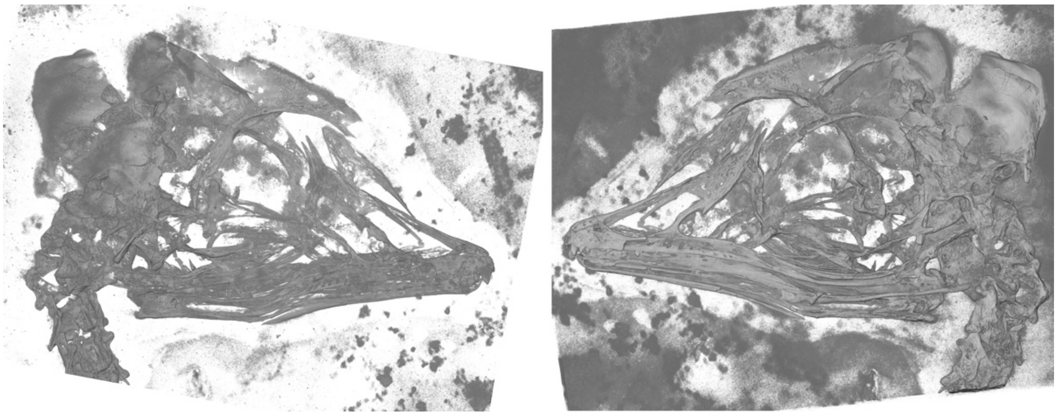

Supplementary Figure 1. Skull of Enantiornithine IVPP V12707. Isosurface of the CT scanning data of the skull.

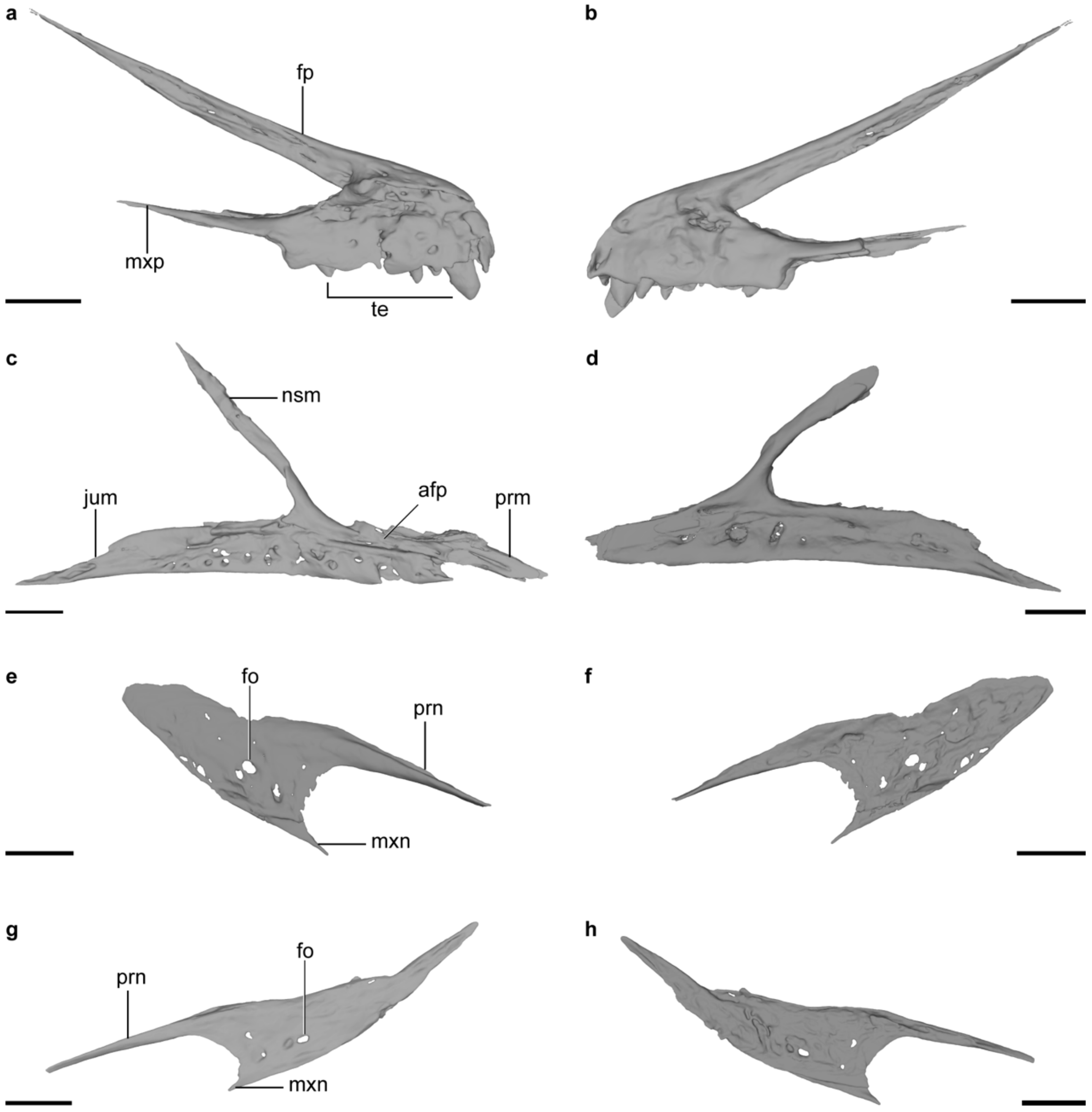

Supplementary Figure 2. Rostral elements of Enantiornithine IVPP V12707. a, b Left (**a**) and right premaxillae (**b**) in lateral view. **c, d** Left (**c**) and right maxillae (**d**) in lateral view. **e, f** Left nasal in lateral (**e**) and medial view (**f**). **g, h** Right nasal in lateral (**g**) and medial view (**h**). afp, articular facet for premaxilla; fp, frontal process of premaxilla; jum, jugal process of maxilla; mxn, maxillary process of nasal; mxp, maxillary process of premaxilla; nsm, nasal process of maxilla; prn, premaxillary process of nasal; te, teeth. Scale bars, 1 mm.

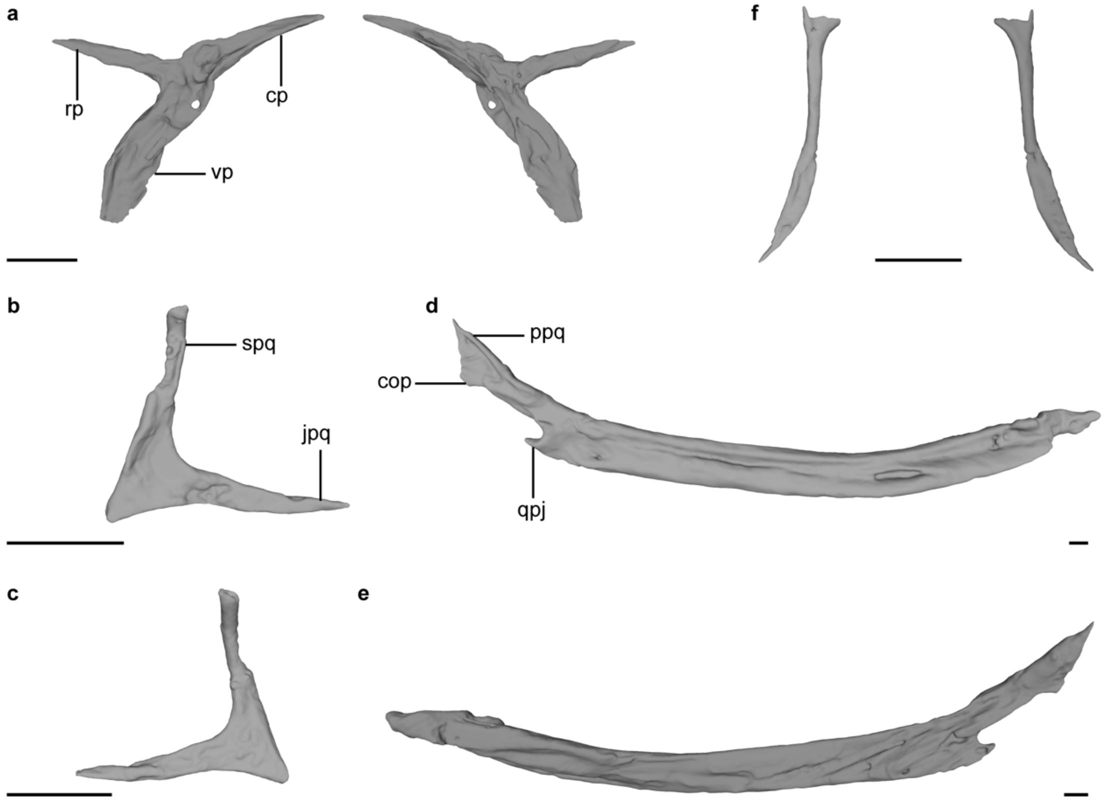

Supplementary Figure 3. Facial elements of IVPP V12707. a Right lacrimal in lateral (left) and medial view (right). **f** Left postorbital in lateral (left) and medial view (right). **b, c** Right quadratojugal in lateral (**b**) and medial view (**c**). **d, e** Right jugal in lateral (**d**) and medial view (**e**). **f** Left postorbital in lateral (left) and medial (right) view. postorbital cop, corneal process; cp, caudal process; fo, foramen; jpq, jugal process of quadratojugal; ppq, postorbital process of jugal; qpj, quadratojugal process of jugal; rp, rostral process; spq, squamosal process of quadratojugal; vp, ventral process. Scale bars, 1 mm.

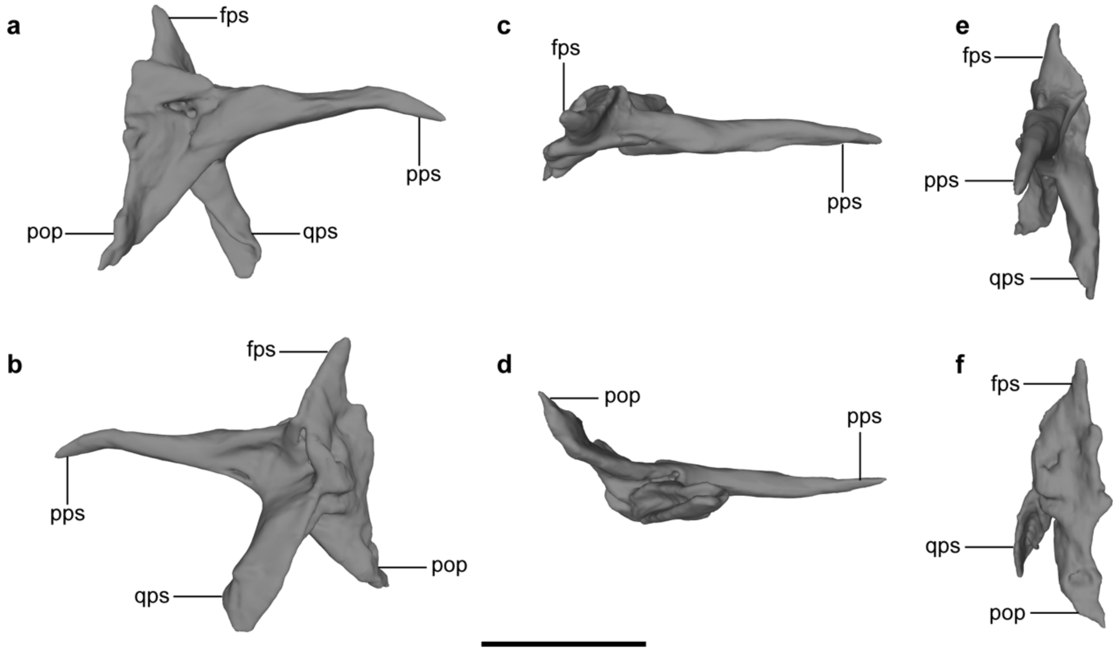

Supplementary Figure 4. Squamosal morphology of IVPP V12707. a–f Right squamosal in lateral (**a**), medial (**b**), dorsal (**c**), ventral (**d**), rostral (**e**), and caudal view (**f**). pap, paroccipital process; pop, postorbital process; prp, parietal processes; qjs, quadratojugal process of squamosal. Scale bars, 10 mm.

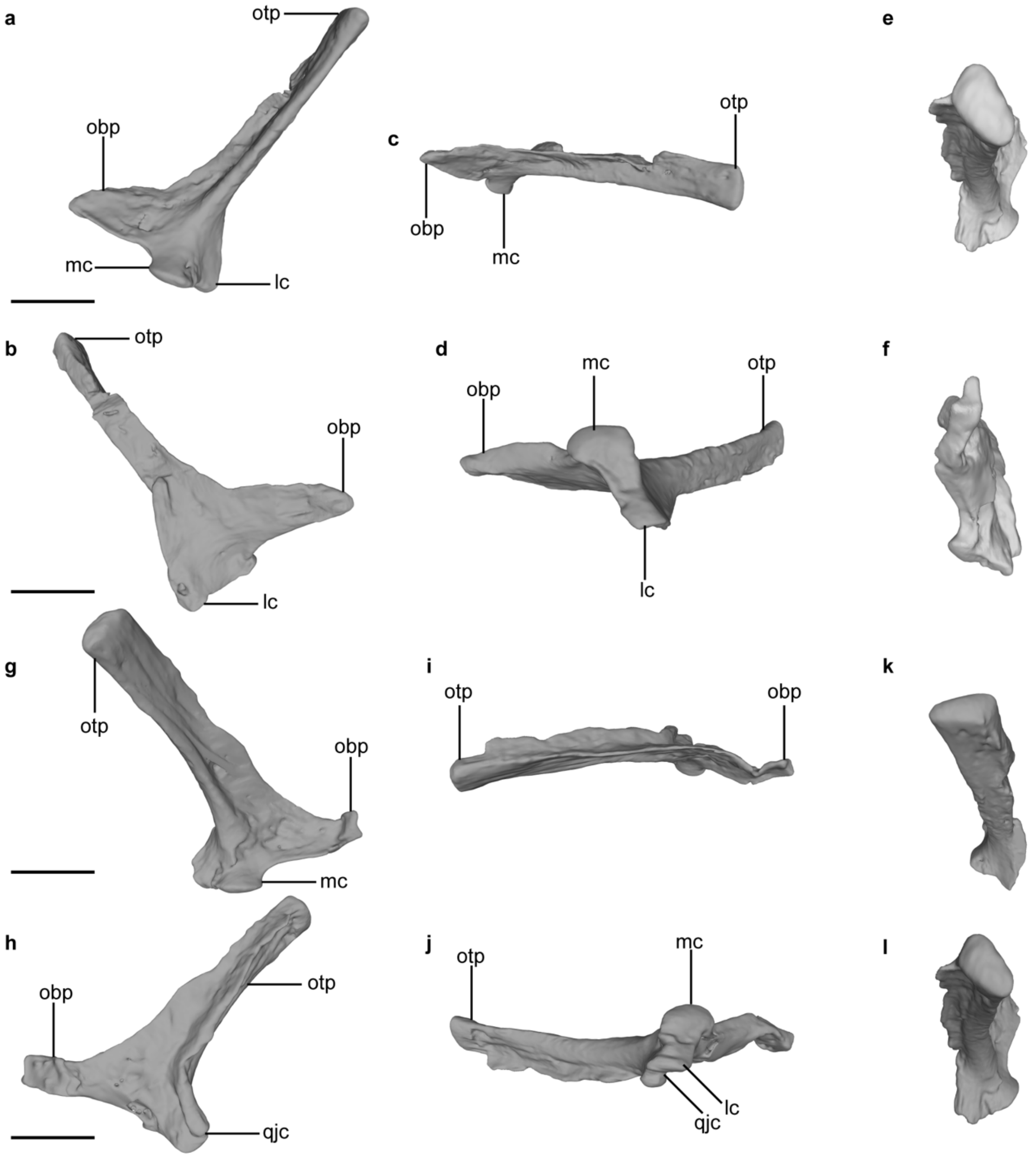

Supplementary Figure 5. Quadrate morphology of IVPP V12707. a–f Right quadrate in caudomedial (**a**), rostrolateral (**b**), dorsal (**c**), ventral (**d**), caudal (**e**), and rostral view (**f**). **g–l** Left quadrate in caudomedial (**g**), rostrolateral (**h**), dorsal (**i**), ventral (**j**), caudal (**k**), and rostral view (**l**). lc, lateral condyle; mc, medial condyle; obp, orbital process; otp, otic process; qjc, quadratojugal cotyla. Scale bars 1 mm.

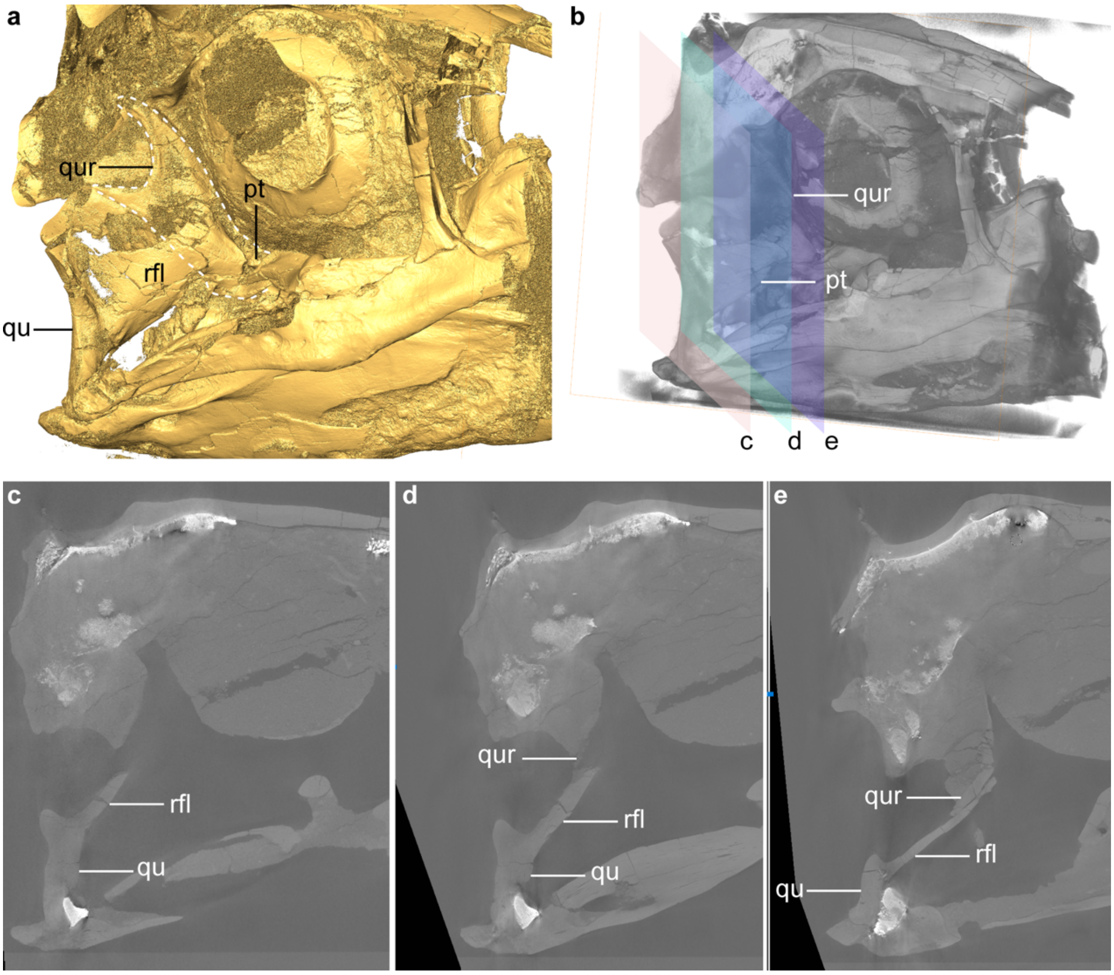

Supplementary Figure 6. Caudal portion of the skull of *Linheraptor* (IVPP V16923). a Isosurface (the white dash line denotes the outline of the quadrate ramus of the pterygoid)**. b** Rendering with slices (**c**–**e**) marked in approximate positions. qu, quadrate; qur, quadrate ramus of the pterygoid; rfl, rostral flange.

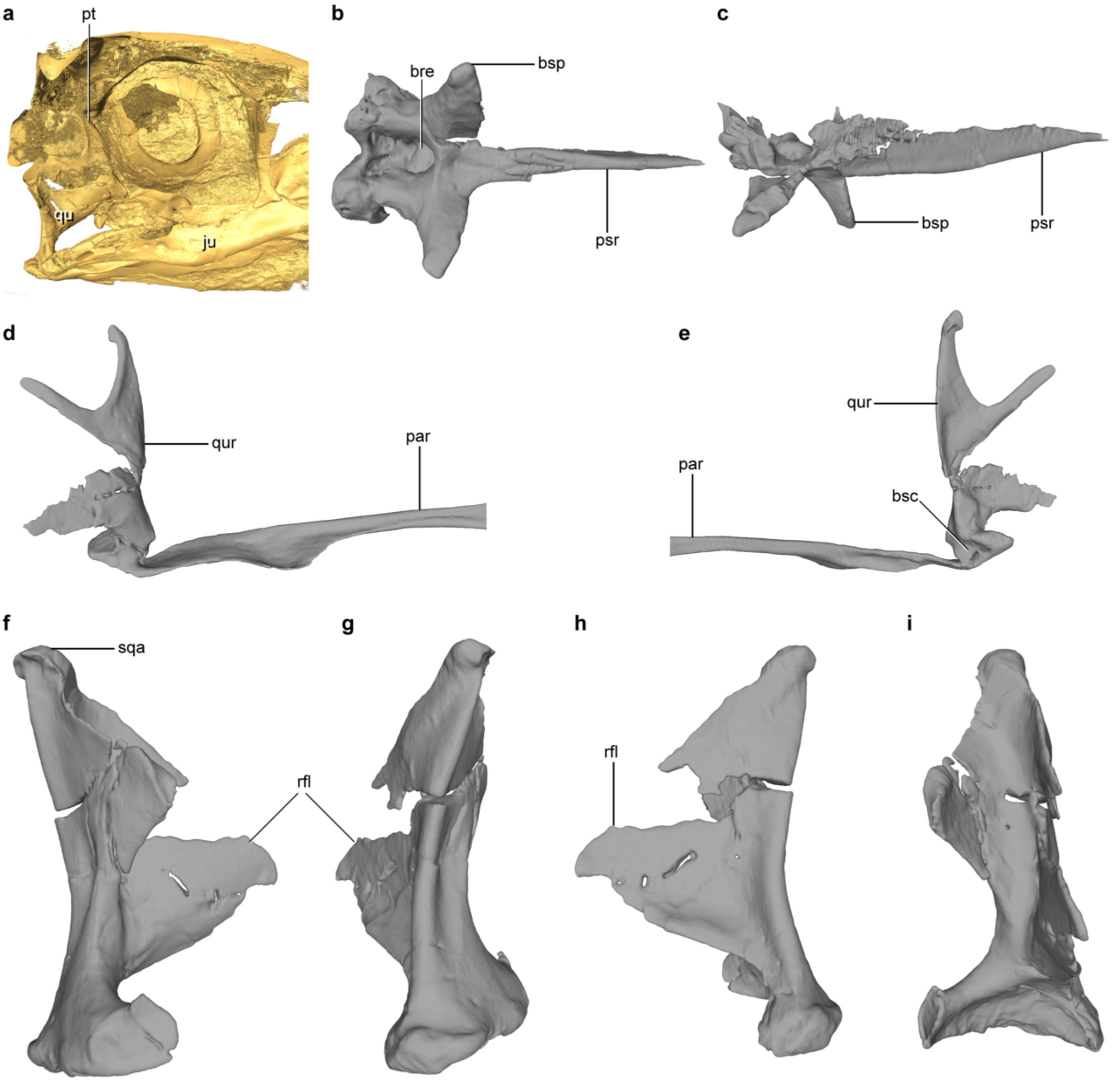

Supplementary Figure 7. Digital reconstruction of palatal elements of *Linheraptor* (IVPP V16923). a Caudal skull in right lateral view. **b, c** Basisphenoid-parasphenoid in ventral (**b**) and rostrolateral view (**c**). **d, e** Right pterygoid in lateral (**d**), and medial view (**e**). **f–i** Right quadrate in lateral (**f**), caudal (**g**), medial (**h**), and rostral view (**i**). bre, bre, basisphenoid recess; bsc, basipterygoid process cotyla; bsp, basipterygoid process; ju, jugal; par, palatine ramus; psr, parasphenoid rostrum; pt, pterygoid; qu, quadrate; qur, quadrate ramus; rfl, rostral flange; sqa, squamosal articular facet.

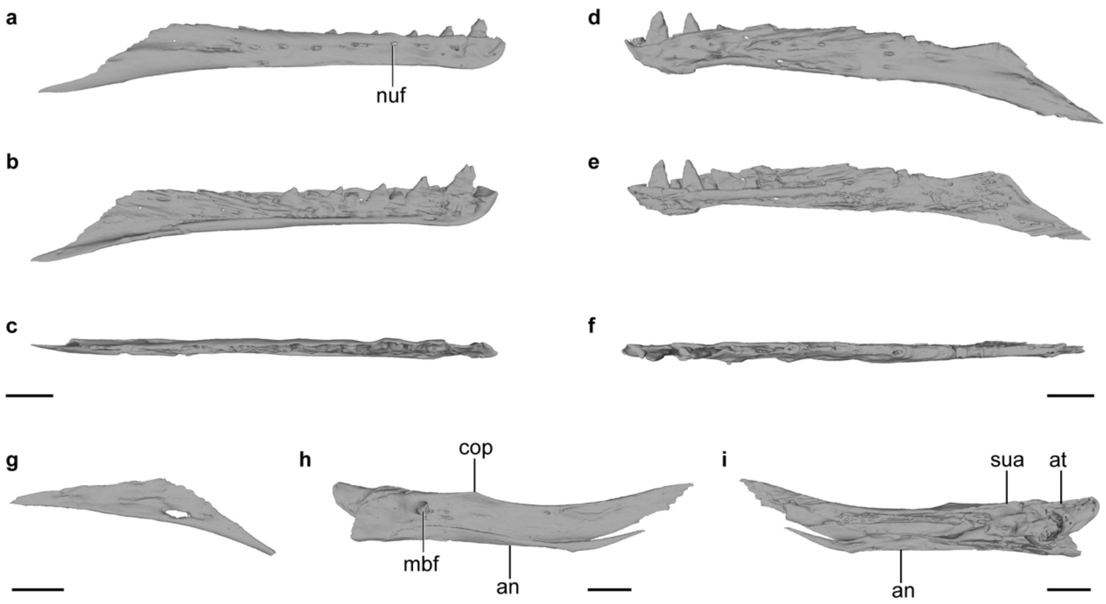

Supplementary Figure 8. Lower jaw elements of IVPP V12707. a–c Right dentary in lateral (**a**), medial (**b**), and dorsal view (**c**). **d–f,** Left dentary in lateral (**d**), medial (**e**), and dorsal view (**f**). **g** Right splenial in medial view. **h, i** Right post-dentary mandibular elements in lateral (**h**) and medial view (**i**). an, angular; at, articular; cop, coronoid process; mbf, mandible foramen; nuf, nutritional foramen; sua, surangular. Scale bars, 1 mm.

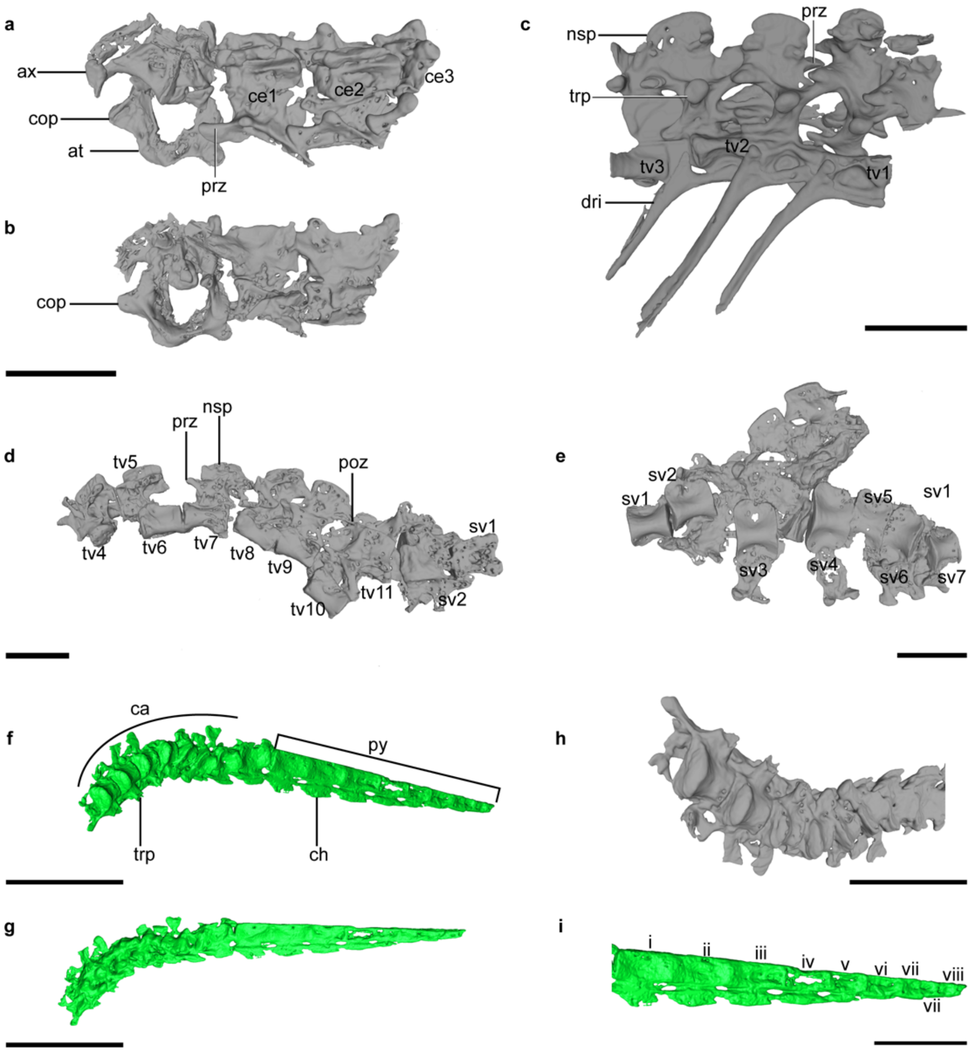

Supplementary Figure 9. Vertebral column of IVPP V12707. a, b Atlas, axis, and cranial-most three cervicals primarily in ventral (**a**) and dorsal view (**b**). **c** Cranial-most three thoracic vertebrae and associated ribs. **d** Caudal eight thoracic vertebrae. **e** Sacral vertebrae primarily in ventral view. **f, g** Caudal vertebrae and pygostyle. **h** Caudal vertebrae in ventral view. **i** Pygostyle in lateral view. at, atlas; ax, axis; ca, caudal vertebrae; ch, chevron; cop, costal process; nsp, neural spine; poz, postzygapophysis; prz, prezygapophysis; py, pygostyle; sv1–7, sacrals vertebrae 1 to 7; trp, transverse process; tv1–11, thoracic vertebrae 1 to 11; i–viii, eight vertebrae incorporated in pygostyle. Scale bars, 0.2 mm (**a–e**), 0.5 mm (**f–i**).

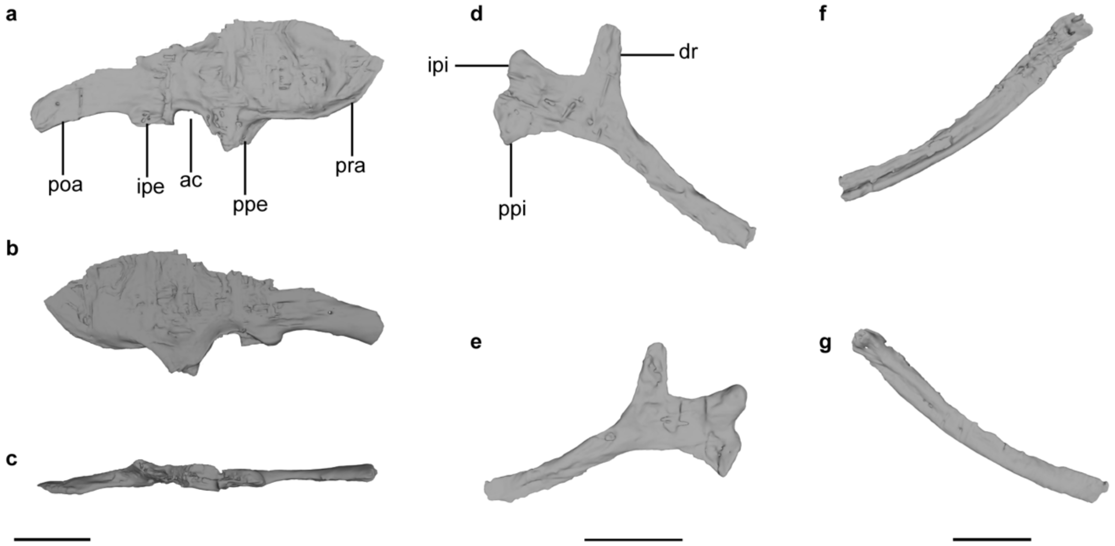

Supplementary Figure 10. Pelvic elements of IVPP V12707. a, b Right ilium in lateral (**a**), medial (**b**), and ventral view (**c**). **d, e** Right ischium in medial (**d**) and lateral view (**e**). **f, g** Right pubis in lateral (**f**) and medial view (**g**). ac, acetabulum; dr, dorsal process; ipe, ischiadic peduncle of ilium; ipi, ischiadic peduncle of ischium; poa, postacetabular process; ppe, pubic peduncle of ilium; ppi, pubic peduncle of ischium; pra, preacetabular process. Scale bars, 2 mm.

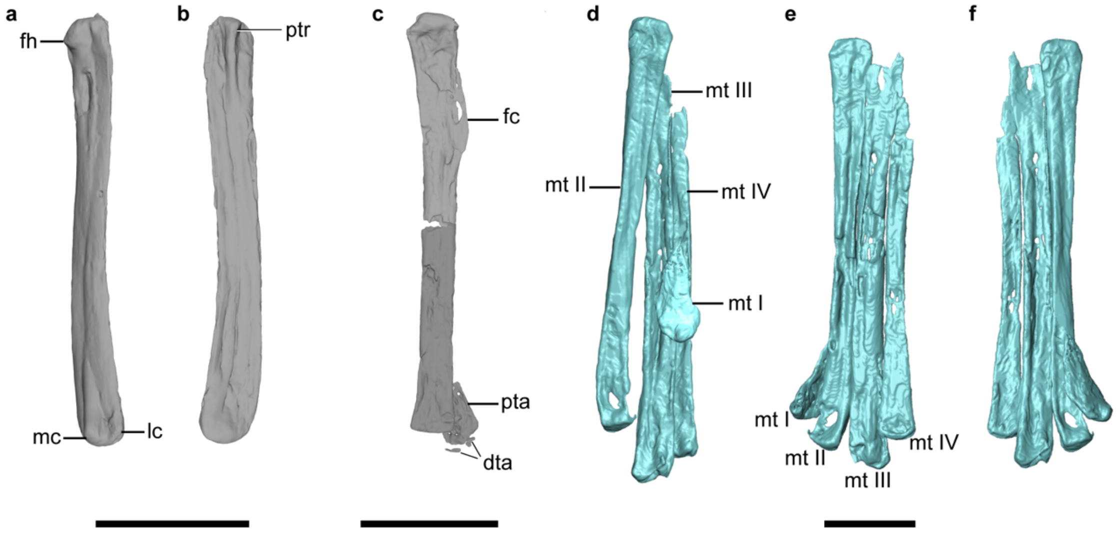

Supplementary Figure 11. Hindlimb of IVPP V12707. a, b Right femur in cranial (**a**) and caudolateral view (**b**). **c** Right tibia in caudal view and associated proximal and distal tarsals. **d** Right metatarsals. **e, f** Digital reconstruction of right metatarsals in ventral (**e**) and dorsal view (**f**). dta, distal tarsals; fc, fibular crest; fh, femoral head; mt I–IV, metatarsals I to IV; pta, proximal tarsals; ptr, posterior trochlea. Scale bars, 5 mm (**a–c**), 2 mm (**e, f**).

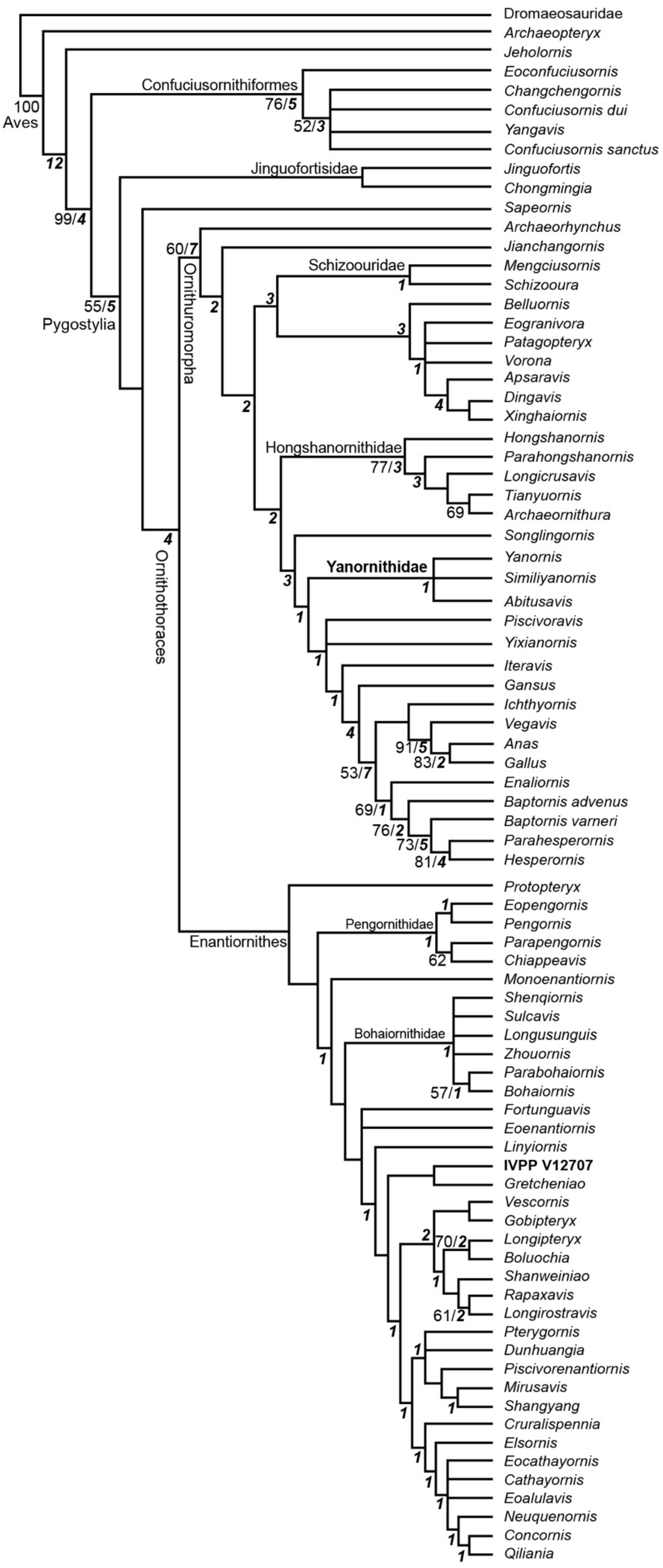

Dromaeosauridae
Archaeopteryx
Jeholornis
Eoconfuciusornis
Changchengornis
Confuciusornis dui
Yangavis
Confuciusornis sanctus
Jinguofortis
Chongmingia
Sapeornis
Archaeorhynchus
Jianchangornis
Mengciusornis
Schizooura
Belluornis
Eogranivora
Patagopteryx
Vorona
Apsaravis
Dingavis
Xinghaiornis
Hongshanornis
Parahongshanornis
Longicrusavis
Tianyuornis
Archaeornithura
Songlingornis
Yanornis
Similiyanornis
Abitusavis
Piscivoravis
Yixianornis
Iteravis
Gansus
Ichthyornis
Vegavis
Anas
Gallus
Enaliornis
Baptornis advenus
Baptornis varneri
Parahesperornis
Hesperornis
Protopteryx
Eopengornis
Pengornis
Parapengornis
Chiappeavis
Monoenantiornis
Shenqiornis
Sulcavis
Longusunguis
Zhouornis
Parabohaiornis
Bohaiornis
Fortunguavis
Eoenantiornis
Linyiornis
IVPP V12707
Gretcheniao
Vescornis
Gobipteryx
Longipteryx
Boluochia
Shanweiniao
Rapaxavis
Longirostravis
Pterygornis
Dunhuangia
Piscivorenantiornis
Mirusavis
Shangyang
Cruralispennia
Elsornis
Eocathayornis
Cathayornis
Eoalulavis
Neuquenornis
Concornis
Qiliania
Aves
Pygostylia
Confuciusornithiformes
Jinguofortisidae
Ornithuromorpha
Ornithothoraces
Schizoouridae
Hongshanornithidae
Yanornithidae
Enantiornithes
Pengornithidae
Bohaiornithidae
100
12
99/4
55/5
76/5
52/3
60/7
4
69
77/3
91/5
83/2
53/7
69/1
76/2
73/5
81/4
62
57/1
70/2
61/2

Supplementary Figure 12. Cladogram of Mesozoic birds showing the position of IVPP V12707. The tree is the strict consensus of the 1344 most parsimonious trees recovered from phylogenetic analysis. The absolute Bootstrap and Bremer values are spell out in normal and bold Italic fonts near corresponding nodes.

Supplementary Note 1

Additional morphological description of the quadrate

As in all enantiornithines[1,2], the quadrate has a bicondylar mandibular process, and the slender otic process is not divided into the squamosal and otic capitula (Fig. 2f; Supplementary Fig. 5). The caudal surface of the bone lacks the foramen of the type present in some enantiornithines[1]. The orbital process is dorsoventrally broad, lacking the tapering shape characteristic of ornithurine birds, *Archaeopteryx*, *Linheraptor*, the troodontid *Mei*, and oviraptorosaurs[3,4]. Instead, the orbital process has a relatively straight and elongate rostrocaudally oriented and thickened ventral edge similar to the condition in *Sapeornis*[5], and the thin rostral margin of the orbital process is broadly concave along its length (in lateral view) possibly as the result of incomplete ossification or preservation of the process. The otic process is more than twice the length of the preserved rostrocaudal length of the orbital process, and it appears that the quadrate was inclined relative to the dorsoventral axis with the otic head positioned caudal to the mandibular condyles, as in some enantiornithines[2]. That slight incline is indicated by the caudal deflection of the mandibular condyles relative to the long axis of the otic process. A fossa is positioned lateral to the base of the orbital process, which

is absent in other non-crown birds such as *Ichthyornis* and *Pterygornis*[2,6].

Supplementary Note 2

Further discussion about the evolution of avian cranial kinesis

Despite the apparent absence of the required cranial kinetic structures and their presumable functions in IVPP V12707, we hypothesize that this individual actually holds a clue related to the evolutionary initiation of kinesis in avialan evolution. Based on our reconstruction, we propose an alternative hypothetical evolutionary pattern and functional scenario for cranial kinesis that is testable with future analyses and data. While the lateral chain of bones and their interconnections appear to restrict the rostrocaudal force transmission and flexibility required for kinesis, it is possible that mobility within the palate and oblique movements may have been the kinetic source material for natural selection. We know among avialans that the pterygoid, palatine, and vomers are interconnected in manners similar to crown birds allowing for force transmission (if present), and that this chain is present in all forms of crown bird kinesis[7,8]. In contrast to these tight osteological interconnections, the morphology of the quadrate in basal avialans with its lack of fusions (streptostyly) and its highly reduced otic head and squamosal articulation with the braincase (reduced even compared to crown birds) would seem to exhibit the features necessary for kinetic movement. While the rostrocaudal movement of the quadrate is key to modern avian kinesis, the kinematics of the avian quadrate also include significant rotation around its dorsoventral long axis[9,10], and that rotation occurs even in taxa with a bifurcated and

expanded otic process of the quadrate. We hypothesize that the quadrate of basal avialans could rotate in such a manner (with the orbital process shifting mediolaterally), and perhaps the highly reduced otic head of the quadrate and its reduced contact with the squamosal facilitated that movement (Fig. 2f; Supplementary Fig. 5). The pterygoid may have worked as a lever within the palate with the basipterygoid processes and their condylar-cotylar contact as the fulcrum. Given the close overlapping association of the orbital process of the quadrate and the ascending quadrate ramus of the pterygoid in avialans and their close dinosaurian outgroups, in what has been termed a scarf joint[11] (Figs. 3, 4), the rotation of the quadrate mediolaterally and its forces would have impacted the ascending ramus of the pterygoid with both structures either acting together as a unit via a joint or through force transmission from the orbital process to the ascending ramus. That rotation and force transmission via the ascending quadrate ramus of the pterygoid would occur at one end of a pterygoid lever with the forces inefficiently transmitted along the pterygoid and rostrally along the palate. The close association and large overlap of the orbital process and ascending ramus (with a great deal of mediolateral overlap see Figs. 3, 4) suggests some functional interaction or grouping between those processes such as a scarf joint present widely among dinosaurs[11]. The transmission of movement from the mobile quadrate to the ascending quadrate ramus of the pterygoid and the rest of the palate could be the evolutionary and functional origin that led under selection to the diversity of kinematics present among crown birds today.

If our analysis is correct and basal birds like enantiornithines were incapable of

kinesis and shared a dinosaurian palate in contrast to their sister group Ornithuromorpha, it is possible that the restructuring of the skull away from this plesiomorphic absence of a pterygoid-quadrate condylar contact and perhaps inefficient transfer of energy between the orbital process of the quadrate and quadrate ramus of the pterygoid in ornithuromorphs led to their greater diversity of skull shapes, diets, and increase in kinetic capabilities. While enantiornithines exhibit some taxa with long rostra and some diversity in skull shape, many early avialan skulls are not as diverse in their shapes. The morphospace occupation of early birds and their interrelated ecologies may have been limited by retained plesiomorphies of the palate and the developmental pathways required to reproduce those structures.

Supplementary Note 3

Dataset used in phylogenetic analysis

The dataset was modified from Wang *et al.*[12] with the addition of IVPP V12707.

Character description:

1. Premaxillae in adults: unfused (0); fused only rostrally (1); completely fused (2). (ordered)
2. Maxillary process of the premaxilla: restricted to its rostral portion (0); subequal or longer than the facial contribution of the maxilla (1).
3. Frontal process of the premaxilla: short (0); relatively long, approaching the rostral border of the antorbital fenestra (1); very long, extending caudally near the level of lacrimals (2).

4. Premaxillary teeth: present throughout (0); present but rostral tip edentulous (1); present but restricted to rostral portion (2); absent (3).

5. Caudal margin of naris: far rostral than the rostral border of the antorbital fossa (0); nearly reaching or overlapping the rostral border of the antorbital fossa (1).

6. Naris longitudinal axis: considerably shorter than the long axis of the antorbital fossa (0); subequal or longer (1).

7. Maxillary teeth: present (0); absent (1).

8. Dorsal (ascending) ramus of the maxilla: present with two fenestrae (the promaxilllary and maxillary fenestra) (0); present with one fenestra (1); infenestrated (2); ramus absent (3). (ordered)

9. Caudal margin of choana: located rostrally, not overlapping the region of the orbit (0); displaced caudally, at the same level or overlapping the rostral margin of the orbit (1).

10. Rostral margin of the jugal: away from the caudal margin of the naris (0); or very close to (leveled with) the caudal margin of the naris (1).

11. Contact between palatine and maxilla/premaxilla: palatine contact maxilla only (0); contacts premaxilla and maxilla (1).

12. Vomer and pterygoid articulation: present, well developed (0); reduced, narrow process of pterygoid passes dorsally over palatine to contact vomer (1); absent, pterygoid and vomer do not contact (2).

13. Jugal process of palatine: present (0); absent (1).

14. Contact between palatine and pterygoid: long, craniocaudally overlapping contact (0); short, primarily dorsoventral contact (1).
15. Contact between vomer and premaxilla: present (0); absent (1).
16. Ectopterygoid: present (0); absent (1).
17. Postorbital: present (0); absent (1).
18. Contact between postorbital and jugal: present (0); absent (1).
19. Quadratojugal: sutured to the quadrate (0); joined through a ligamentary articulation (1).
20. Lateral, round cotyla on the mandibular process of the quadrate (quadratojugal articulation): absent (0); present (1).
21. Contact between the quadratojugal and squamosal: present (0); absent (1).
22. Squamosal incorporated into the braincase, forming a zygomatic process: absent (0); present (1).
23. Squamosal, ventral or "zygomatic" process: variably elongate, dorsally enclosing otic process of the quadrate and extending cranioventrally along shaft of this bone, dorsal head of quadrate not visible in lateral view (0); short, head of quadrate exposed in lateral view (1).
24. Frontal/parietal suture in adults: open (0); fused (1).
25. Quadrate orbital process (pterygoid ramus): broad (0); sharp and pointed (1).
26. Quadrate pneumaticity: absent (0); present (1).
27. Quadrate: articulating only with the squamosal (0); articulating with both prootic and squamosal (1).

28. Otic articulation of the quadrate: articulates with a single facet (squamosal) (0); articulates with two distinct facets (prootic and squamosal) (1); articulates with two distinct facets and quadrate differentiated into two heads (2). (ordered)

29. Quadrate distal end: with two transversely aligned condyles (0); with a triangular, condylar pattern, usually composed of three distinct condyles (1).

30. Basipterygoid processes: long (0); short (articulation with pterygoid subequal to, or longer than, amount projected from the basisphenoid rostrum) (1).

31. Pterygoid, articular surface for basipterygoid process: concave "socket", or short groove enclosed by dorsal and ventral flanges (0); flat to convex (1); flat to convex facet, stalked, variably projected (2). (ordered)

32. Eustachian tubes: paired, lateral, and well-separated from each other (0); paired, close to each other and to cranial midline or forming a single cranial opening (1).

33. Osseous interorbital septum (mesethmoid): absent (0); present (1).

34. Dentary teeth: present (0); absent (1).

35. Dentary tooth implantation: teeth in individual sockets (0); teeth in a communal groove (1).

36. Symphysial portion of dentaries: unfused (0); fused (1).

37. Deeply notched rostral end of the mandibular symphysis: absent (0); present (1).

38. Mandibular symphysis, symphyseal foramina: absent (0); single (1); paired (2).

39. Mandibular symphysis, symphyseal foramen/foramina: opening on caudal edge of symphysis (0); opening on dorsal surface of symphysis (1).

40. Small ossification present at the rostral tip of the mandibular symphysis

(intersymphysial ossification): absent (0); present (1).

41. Caudal margin of dentary strongly forked: unforked, or with a weakly developed dorsal ramus (0); strongly forked with the dorsal and ventral rami approximately equal in caudal extent (1).
42. Mandibular ramus sigmoidal such that the rostral tip is dorsally convex and the caudal end is dorsally concave: absent (0); present (1).
43. Cranial extent of splenial: stops well caudal to mandibular symphysis (0); extending to mandibular symphysis, though non-contacting (1); extending to proximal tip of mandible, contacting on midline (2). (ordered)
44. Meckel's groove (medial side of mandible): not completely covered by splenial, deep and conspicuous medially (0); covered by splenial, not exposed medially (1).
45. Rostral mandibular fenestra: absent (0); present (1).
46. Caudal mandibular fenestra: present (0); absent (1).
47. Articular pneumaticity: absent (0); present (1).
48. Teeth: serrated crowns (0); unserrated crowns (1).
49. Atlantal hemiarches in adults: unfused (0); fused, forming a single arch (1).
50. One or more pneumatic foramina piercing the centra of mid-cranial cervicals, caudal to the level of the parapophysis-diapophysis: present (0); absent (1).
51. Cervical vertebrae: variably dorsoventrally compressed, amphicoelous ("biconcave": flat to concave articular surfaces) (0); cranial surface heterocoelous (i.e., mediolaterally concave, dorsoventrally convex), caudal surface flat or slightly concave (1); heterocoelous cranial (i.e., mediolaterally concave, dorsoventrally

convex) and caudal (i.e., mediolaterally convex, dorsoventrally concave) surfaces (2). (ordered)

52. Prominent carotid processes in the intermediate cervicals: absent (0); present (1).

53. Postaxial cervical epipophyses: prominent, projecting further back from the postzygapophysis (0); weak, not projecting further back from the postzygapophysis, or absent (1).

54. Keel-like ventral surface of cervical centra: absent (0); present (1).

55. Prominent (50% or more the height of the centrum's cranial articular surface) ventral processes of the cervicothoracic vertebrae: absent (0); present (1).

56. Thoracic vertebral count: 13-14 (0); 11-12 (1); fewer than 11 (2). (ordered)

57. Thoracic vertebrae: at least part of series with subround, central articular surfaces (e.g., amphicoelous/opisthocoelous) that lack the dorsoventral compression seen in heterocoelous vertebrae (0); series completely heterocoelous (1).

58. Caudal thoracic vertebrae, centra, length and midpoint width: approximately equal in length and midpoint width (0); length markedly greater than midpoint width (1).

59. Wide vertebral foramen in the mid-caudal thoracic vertebrae, vertebral foramen/articular cranial surface ratio (vertical diameter) larger than 0.40: absent (0); present (1).

60. Hyposphene-hypantrum accessory intervertebral articulations in the thoracic vertebrae: present (0); absent (1).

61. Lateral side of the thoracic centra: weakly or not excavated (0); deeply excavated

by a groove (1); excavated by a broad fossa (2).

62. Cranial thoracic vertebrae, parapophyses: located in the cranial part of the centra of the thoracic vertebrae (0); located in the central part of the centra of the thoracic vertebrae (1).

63. Notarium: absent (0); present (1).

64. Sacral vertebrae, number ankylosed (synsacrum): less than 7 (0); 7 (1); 8 (2); 9 (3); 10 (4); 11 or more (5); 15 or more (6). (ordered)

65. Synsacrum, procoelous articulation with last thoracic centrum (deeply concave facet of synsacrum receives convex articulation of last thoracic centrum): absent (0); present (1).

66. Cranial vertebral articulation of first sacral vertebra: approximately equal in height and width (0); wider than high (1).

67. Series of short sacral vertebrae with dorsally directed parapophyses just cranial to the acetabulum: absent (0); present, three such vertebrae (1); present, four such vertebrae (2). (ordered)

68. Convex caudal articular surface of the synsacrum: absent (0); present (1).

69. Degree of fusion of distal caudal vertebrae: fusion absent (0); few vertebrae partially ankylosed (intervening elements are well-discernable) (1); vertebrae completely fused into a pygostyle (2). (ordered)

70. Free caudal vertebral count: more than 35 (0); 35-26 (1); 25 - 20 (2); 19-9 (3); 8 or less (4). (ordered)

71. Procoelous caudals: absent (0); present (1).

72. Distal caudal vertebra prezygapophyses: elongate, exceeding the length of the centrum by more than 25% (0); shorter (1); absent (2). (ordered)

73. Free caudals, length of transverse processes: approximately equal to, or greater than, centrum width (0); significantly shorter than centrum width (1).

74. Proximal haemal arches: elongate, at least 3 times longer than wider (0); shorter (1); absent (2). (ordered)

75. Pygostyle: longer than or equal to the combined length of the free caudals (0); shorter (1).

76. Cranial end of pygostyle dorsally forked: absent (0); present (1).

77. Cranial end of pygostyle with a pair of laminar, ventrally projected processes: absent (0); present (1).

78. Distal constriction of pygostyle: absent (0); present (1).

79. Ossified uncinate processes in adults: absent (0); present and free (1); present and fused (2).

80. Uncinate process, orientation: perpendicular to rib (0); angled dorsally defining an acute angle with the rib (1).

81. Gastralia: present (0); absent (1).

82. Coracoid shape: rectangular to trapezoidal in profile (0); strut-like (1).

83. Coracoid and scapula articulation: pit-shaped scapular cotyla developed on the coracoid, and coracoidal tubercle developed on the scapula ("ball and socket" articulation) (0); scapular articular surface of coracoid convex (1); (2) flat.

84. Scapula: articulated at the shoulder (proximal) end of the coracoid (0); well below it (1).

85. Coracoid, humeral articular (glenoid) facet: dorsal to acrocoracoid process/"biceps tubercle" (0); ventral to acrocoracoid process (1).

86. Humeral articular facets of the coracoid and the scapula: placed in the same plane (0); forming a sharp angle (1).

87. Coracoid, acrocoracoid: straight (0); hooked medially (1).

88. Laterally compressed shoulder end of coracoid, with nearly aligned acrocoracoid process, humeral articular surface, and scapular facet, in dorsal view: absent (0); present (1).

89. Procoracoid process on coracoid: absent (0); present (1).

90. Lateral margin of coracoid: concave (0); nearly concave to straight for most part and the convex portion is restricted at sternal end, which measures less than half the width of sternal end (1); strongly convex, and the convex portion measuring more than half the sternal end (2).

91. Broad, deep fossa on the dorsal surface of the coracoid (dorsal coracoidal fossa): absent (0); present (1).

92. Supracoracoidal nerve foramen of coracoid: centrally located (0); displaced toward (often as an incisure) the medial margin of the coracoid (1); displaced so that it no longer passes through the coracoid (absent) (2). (ordered).

93. Coracoid, medial surface, strongly depressed elongate furrow at the level of the passage of n. supracoracoideus: absent (0); present (1).

94. Supracoracoidal nerve foramen, location relative to dorsal coracoidal fossa: above fossa (0); inside fossa (1).

95. Coracoid, sternolateral corner: unexpanded (0); expanded (1); well-developed squared-off lateral process (sternocoracoidal process) (2); present and with a distinct omal projection (hooked) (3).

96. Scapular shaft: straight, both dorsal and ventral margins straight (0); straight shaft with convex dorsal margin and straight ventral margin (1); the scapular shaft sagittally curved (2).

97. Scapula, length: shorter than humerus (0); as long as or longer than humerus (1).

98. Scapular acromion process: in lateral or costal view, strongly projecting craniodorsally, forming a large angle with the proximal shaft of the scapular (0); nearly parallel to the shaft of the scapular (1).

99. Scapula, acromion process: projected cranially surpassing the articular surface for coracoid (0); projected less cranially than the articular surface for coracoid (1).

100. Scapula, acromion process, in costal or lateral aspect: straight and tapered toward cranial end (0); barely tapered with a blunt end (1); laterally hooked tip (2).

101. Proximal end of scapula, pit between acromion and humeral articular facet (scapular fossa): absent (0); present (1).

102. Costal surface of scapular blade with prominent longitudinal furrow: absent (0); present (1).

103. Scapular caudal end: blunt (may or may not be expanded) (0); sharply tapered (1).

104. Furcular, shape: boomerang-shaped (0); V to Y-shaped (1); U-shaped (2).

105. Furcula interclavicular angle: approximately 90° (0); less than 70° (1). The interclavicular angle is measured as the angle formed between three points, one at the omal end of each rami and the apex located at the clavicular symphysis.

106. Dorsal and ventral margins of the furcula: subequal in width (0); ventral margin distinctly wider than the dorsal margin so that the furcular ramus appears concave laterally (1).

107. Hypocleideum: absent (0); present as a tubercle or short process (1); present as an elongate process approximately 30% rami length (2); hypertrophied, exceeding 50% rami length (3). (ordered)

108. Sternum: unossified (0); partially ossified, coracoidal facets cartilaginous (1); fully ossified (2).

109. Ossified sternum: two flat plates (0); single flat element (1); single element, with slightly raised midline ridge (2); single element, with projected carina (3).

110. Sternal carina: near to, or projecting rostrally from, the cranial border of the sternum (0); not reaching the cranial border of the sternum (1).

111. Sternum, caudal margin, number of paired caudal trabecula: none (0); one (1); two (2).

112. Sternum, outermost trabecula, shape: tips terminate cranial to caudal end of sternum (0); tips terminate at or approaching caudal end of sternum (1); tips extend caudally past the termination of the sternal midline (2).

113. Prominent distal expansion in the outermost trabecula of the sternum: absent (0);

present, simple bulb-like (1); fan-shaped expansion (2); triangular expansion with an acute medial angle (3); branched (4).

114. Rostral margin of the sternum broad and rounded: absent (0); present (1).

115. Sternum, coracoidal sulci spacing on cranial edge: widely separated mediolaterally (0); adjacent (1); crossed on midline (2).

116. Costal facets of the sternum: absent (0); present (1).

117. Sternal costal processes: three (0); four (1); five (2); six (3); seven (4); eight (5). (ordered)

118. Sternal midline, caudal end: blunt W-shape (0); V-shape (1); elongate straight projection (xiphoid process) (2); xiphoid process slightly flared mediolaterally (3); xiphoid process distal end strongly flared with prominent medial and lateral projections (4); rounded (5).

119. Sternum, caudal half, paired enclosed fenestra: absent (0); present (1).

120. Sternum, dorsal surface, pneumatic foramen (or foramina): absent (0); present (1).

121. Proximal and distal humeral ends: twisted (0); expanded nearly in the same plane (1).

122. Humeral head: concave cranially and convex caudally (0); globe shaped, craniocaudally convex (1).

123. Proximal margin of the humeral head concave in its central portion, rising ventrally and dorsally: absent (0); present (1).

124. Humerus, proximocranial surface, well-developed circular fossa on midline: absent (0); present (1).

125. Humerus with distinct transverse ligamental groove: absent (0); present (1).

126. Humerus, ventral tubercle projected caudally, separated from humeral head by deep capital incision: absent (0); present (1).

127. Pneumatic fossa in the caudoventral corner of the proximal end of the humerus: absent or rudimentary (0); well developed (1).

128. Humerus, deltopectoral crest: projected dorsally (the plane of the crest is coplanar to the cranial surface of the humerus) (0); projected cranially (1).

129. Humerus, deltopectoral crest: less than shaft width (0); approximately same width (1); prominent and subquadrangular (i.e., subequal length and width) (2).

130. Humerus, deltopectoral crest, perforated by a large fenestra: absent (0); present (1).

131. Humerus, bicipital crest: little or no cranial projection (0); developed as a cranial projection relative to shaft surface in ventral view (1); hypertrophied, rounded tumescence (2).

132. Humerus, distal end of bicipital crest, pit-shaped fossa for muscular attachment: absent (0); craniodistal on bicipital crest (1); directly ventrodistal at tip of bicipital crest (2); caudodistal, variably developed as a fossa (3).

133. Distal end of the humerus very compressed craniocaudally: absent (0); present (1).

134. Humerus, demarcation of muscle origins (e.g., m. extensor metacarpi radialis in Aves) on the dorsal edge of the distal humerus: no indication (0); a pit or a

tubercle (1); a variably projected scar-bearing tubercle (dorsal supracondylar process) (2).

135. Well-developed brachial depression on the cranial face of the distal end of the humerus: absent (0); present (1).

136. Well-developed olecranon fossa on the caudal face of the distal end of the humerus: absent (0); present (1).

137. Humerus, distal end, caudal surface, groove for passage of m. scapulotriceps: absent (0); present (1).

138. Humerus, m. humerotricipitalis groove: absent (0); present as a well-developed ventral depression contiguous with the olecranon fossa (1).

139. Humerus, distal margin: approximately perpendicular to long axis of humeral shaft (0); ventrodistal margin projected significantly distal to dorsodistal margin, distal margin angling strongly ventrally (sometimes described as a well-projected flexor process) (1).

140. Humeral distal condyles: mainly located on distal aspect (0); on cranial aspect (1).

141. Humerus, long axis of dorsal condyle: at low angle to humeral axis, proximodistally oriented (0); at high angle to humeral axis, almost transversely oriented (1).

142. Humerus, distal condyles: subround, bulbous (0); weakly defined, “straplike” (1).

143. Humerus, ventral condyle: length of long axis of condyle less than the same measure of the dorsal condyle (0); same or greater (1).

144. Ulna: shorter than humerus (0); nearly equivalent to or longer than humerus (1).

145. Ulnar shaft, radial-shaft/ulnar-shaft ratio: larger than 0.70 (0); smaller than 0.70 (1).

146. Ulna, cotylae: dorsoventrally adjacent (0); widely separated by a deep groove (1).

147. Ulna, dorsal cotyla strongly convex: absent (0); present (1).

148. Ulna, bicipital scar: absent (0); developed as a slightly raised scar (1); developed as a conspicuous tubercle (2).

149. Proximal end of the ulna with a well-defined area for the insertion of m. brachialis anticus: absent (0); present (1).

150. Semilunate ridge on the dorsal condyle of the ulna: absent (0); present (1).

151. Shaft of radius with a long longitudinal groove on its ventrocaudal surface: absent (0); present (1).

152. Ulnare: heart-shaped with little differentiation into short rami (0); U-shaped to V-shaped, well-developed rami (1).

153. Ulnare, ventral ramus: shorter than dorsal ramus (crus brevis) (0); same length as dorsal ramus (1); longer than dorsal ramus (2).

154. Semilunate carpal and proximal ends of metacarpals in adults: unfused (0); semilunate fused to the alular (I) metacarpal (1); semilunate fused to the major (II) and minor (III) metacarpals (2); fusion of semilunate and all metacarpals (3). Any specimen that is inferred to be a juvenile should be scored as a "?" in order to account for the possibility of ontogenetic change.

155. Semilunate carpal, position relative to the alular metacarpal (I): over entire proximal surface (0); over less than one-half proximal surface or no contact present (1).

156. Carpometacarpus, proximal ventral surface: flat (0); raised ventral projection contiguous with minor metacarpal (1); pisiform process forming a distinct peg-like projection (2).

157. Carpometacarpus, ventral surface, supratrochlear fossa deeply excavating proximal surface of pisiform process: absent (0); present (1).

158. Round-shaped alular metacarpal (I): absent (0); present (1).

159. Alular metacarpal (I), extensor process: absent, no cranioproximally projected muscular process (0); present, tip of extensor process just surpassed the distal articular facet for phalanx 1 in cranial extent (1); tip of extensor process conspicuously surpasses articular facet by approximately half the width of facet, producing a pronounced knob (2); tip of extensor process conspicuously surpasses articular facet by approximately the width of facet, producing a pronounced knob (3). (ordered)

160. Alular metacarpal (I), distal articulation with phalanx I: ginglymoid (0); shelf (1); ball-like (2).

161. Metacarpal III, craniocaudal diameter as a percentage of same dimension of metacarpal II: approximately equal or greater than 50% (0); less than 50% (1).

162. Proximal extension of metacarpal III: level with metacarpal II (0); ending distal to proximal surface of metacarpal II (1).

163. Intermetacarpal process or tubercle on metacarpal II: absent (0); present as scar (1); present as tubercle or flange (2).

164. Intermetacarpal space: absent or very narrow (0); at least as wide as the maximum width of minor metacarpal (III) shaft (1).

165. Intermetacarpal space: reaches proximally as far as the distal end of metacarpal I (0); terminates distal to end of metacarpal I (1).

166. Distal end of metacarpals: unfused (0); partially or completely fused (1).

167. Minor metacarpal (III) projecting distally more than the major metacarpal (II): absent (0); present (1).

168. Alular digit (I), phalanx 1, distal extension relative to the major metacarpal (II): beyond the distal end of major metacarpal (0); approximately equal in distal extension (1); shorter than the distal end but beyond half of the major metacarpal (2); terminating less than half of the major metacarpal (3). (ordered)

169. Proximal phalanx of major digit (II): of normal shape (0); flat and craniocaudally expanded (1).

170. Major digit (II), phalanx 1, "internal index process" (Stegmann, 1978) on caudodistal edge: absent (0); present (1).

171. Second phalanx of major digit (II): longer than proximal phalanx (0); shorter than or equivalent to proximal phalanx (1).

172. Ungual phalanx of major digit (II): present (0); absent (1).

173. Ungual phalanx of major digit (II): larger or subequal to other manual unguals (0); smaller than the alular ungual but larger than that of the minor (III) digit, and the

ungual of the minor digit may or may not present (1); smaller than the unguals of the alular and minor digits (2).

174. Proximal phalanx of the minor digit (III) much shorter than the remaining non-ungual phalanges of this digit: absent (0); present (1).

175. Ungual phalanx of minor digit (III): present (0); absent (1).

176. Length of manus (semilunate carpal + major metacarpal and digit) relative to humerus: longer (0); subequal (1); shorter (2). (ordered)

177. Intermembral index = (length of humerus + ulna)/(length of femur + tibiotarsus): less than 0.7, flightless (0); between 0.7 and 0.9 (1); between 0.9 and 1.1 (2); greater than 1.1 (3).

178. Pelvic elements in adults, at the level of the acetabulum: unfused or partial fusion (0); completely fused (1).

179. Ilium/ischium, distal co-ossification to completely enclose the ilioischiadic fenestra: absent (0); present (1).

180. Preacetabular process of ilium twice as long as postacetabular process: absent (0); present (1).

181. Preacetabular ilium: approach on midline, open, or cartilaginous connection (0); co-ossified, dorsal closure of “iliosynsacral canals” (1).

182. Ilium, m. cuppedicus fossa as broad, mediolaterally oriented surface directly cranioventral to acetabulum: present (0); surface absent, insertion variably marked by a small entirely lateral fossa cranial to acetabulum (1).

183. Preacetabular pectineal process: absent (0); present as a small flange (1); present as a well-projected flange (2). (ordered)

184. Small acetabulum, acetabulum/ilium length ratio equal to or smaller than 0.11: absent (0); present (1).

185. Prominent antitrochanter: caudally directed (0); caudodorsally directed (1).

186. Postacetabular process shallow, less than 50% of the depth of the preacetabular wing at the acetabulum: absent (0); present (1).

187. Iliac brevis fossa: present (0); absent (1).

188. Ischium: two-thirds or less the length of the pubis (0); more than two-thirds the length of the pubis (1).

189. Obturator process of ischium: prominent (0); reduced or absent (1).

190. Ischium, caudal demarcation of the obturator foramen: absent (0); present, developed as a small flange or raised scar contacting/fused with pubis and demarcating the obturator foramen distally (1).

191. Ischium with a proximodorsal (or proximocaudal) process: absent (0); present (1).

192. Ischiadic terminal processes forming a symphysis: present (0); absent (1).

193. Orientation of proximal portion of pubis: cranially to subvertically oriented (0); retroverted, separated from the main synsacral axis by an angle ranging between 65º and 45º (1); more or less parallel to the ilium and ischium (2). (ordered)

194. Pubic pedicel: cranioventrally projected (0); ventrally or caudoventrally projected (1).

195. Pubic pedicel of ilium very compressed laterally and hook-like: absent (0), present

(1).

196. Pubic shaft laterally compressed throughout its length: absent (0); present (1).

197. Pubic apron: present (0); absent (absence of symphysis) (1).

198. Pubic foot: flaring into simple round shape (0); triangular shape with a pointed caudal tip and caudoventrally directed with respect to the distal pubic shaft (1); the caudal tip recurved caudodorsally with respect to the distal pubic shaft (2); absent (3).

199. Femur with distinct fossa for the capital ligament: absent (0); present (1).

200. Femoral neck: present (0); absent (1).

201. Femoral anterior trochanter: separated from the greater trochanter (0); fused to it, forming a trochanteric crest with a laterally curved edge (1); fused to it, forming a trochanteric crest with a flattened edge (2).

202. Femoral trochanteric crest: projects proximally beyond femoral head (0); equal in proximal projection (1); does not project beyond femoral head (2).

203. Femoral posterior trochanter: present, developed as a slightly projected tubercle or flange (0); hypertrophied, “shelf-like” conformation (1); absent (2).

204. Femur with prominent patellar groove: absent (0); present as a continuous extension onto the distal shaft (1); present and separated from the shaft by a slight ridge, giving it a pocketed appearance (2).

205. Femur: ectocondylar tubercle and lateral condyle separated by deep notch (0); ectocondylar tubercle and lateral condyle contiguous but without developing a tibiofibular crest (1); tibiofibular crest present, defining laterally a fibular trochlea

(2). (ordered)

206. Caudal projection of the lateral border of the distal end of the femur, proximal and contiguous to the ectocondylar tubercle/tibiofibular crest: absent (0); present (1).

207. Femoral popliteal fossa distally bounded by a complete transverse ridge: absent (0); present (1).

208. Fossa for the femoral origin of m. tibialis cranialis: absent (0); present (1).

209. Tibia, calcaneum, and astragalus: unfused or poorly co-ossified (sutures still visible) (0); complete fusion of tibia, calcaneum, and astragalus (1).

210. Round proximal articular surface of tibiotarsus: absent (0); present (1).

211. Tibiotarsus, proximal articular surface: flat (0); angled so that the medial margin is elevated with respect to the lateral margin (1).

212. Tibiotarsus, cnemial crests: absent (0); present, one (1); present, two (2).

213. Tibia, caudal extension of articular surface for distal tarsals/tarsometatarsus: absent, articular restricted to distalmost edge of caudal surface (0); well-developed caudal extension, sulcus cartilaginis tibialis of Aves, distinct surface extending up the caudal surface of the tibiotarsus (1); with well-developed, caudally projecting medial and lateral crests (2). (ordered)

214. Extensor canal on tibiotarsus: absent (0); present as an emarginate groove (1); groove bridged by an ossified supratendinal bridge (2). (ordered)

215. Tibia/tarsal-formed condyles: medial condyle projecting farther cranially than lateral condyle (0); equal in cranial projection (1).

216. Tibia/tarsal-formed condyles, mediolateral widths: medial condyle wider (0); approximately equal (1); lateral condyle wider (2). (ordered).

217. Tibia/tarsal-formed condyles: gradual sloping of condyles towards midline of tibiotarsus (0); no tapering of either condyle (1).

218. Proximal end of the fibula: prominently excavated by a medial fossa (0); nearly flat (1).

219. Fibula, tubercle for m. iliofibularis: craniolaterally directed (0); laterally directed (1); caudolaterally or caudally directed (2). (ordered)

220. Fibula, distal end reaching the proximal tarsals: present (0); absent (1).

221. Distal tarsals in adults: free (0); completely fused to the metatarsals (1). Any specimen that is inferred to be a juvenile should be scored as a "?" in order to account for the possibility of ontogenetic change.

222. Metatarsals II-IV, intermetatarsal fusion: absent or minimal co-ossification (0); partial fusion, sutural contacts easily discernible (1); completely or nearly completely fused, sutural contacts absent or poorly demarcated (2). (ordered)

223. Proximal end of metatarsus: plane of articular surface perpendicular to longitudinal axis of metatarsus (0); strongly inclined dorsally (1).

224. Metatarsal V: present (0); absent (1).

225. Proximal end of metatarsal III: in the same plane as metatarsals II and IV (0); plantarly displaced with respect to metatarsals II and IV (1).

226. Tarsometatarsal proximal vascular foramen/foramina: absent (0); one between metatarsals III and IV (1); two (2).

227. Metatarsals, relative mediolateral width: metatarsal IV approximately the same width as metatarsals II and III (0); metatarsal IV narrower than metatarsals II and III (1); metatarsal IV greater in width than either metatarsal II or III (2).

228. Well-developed tarsometatarsal intercotylar eminence: absent (0); present, low and rounded (1); present, high and peaked (2).

229. Tarsometatarsus, projected surface and/or grooves on proximocaudal surface (associated with the passage of tendons of the pes flexors in Aves; hypotarsus): absent (0); developed as caudal projection with flat caudal surface (1); projection, with distinct crests and grooves (2); at least one groove enclosed by bone caudally (3). (ordered)

230. Plantar surface of tarsometatarsus excavated: absent (0); present (1).

231. Tarsometatarsal distal vascular foramen completely enclosed by metatarsals III and IV: absent (0); present (1).

232. Metatarsal I: straight (0); J-shaped, the articulation of the hallux is located on the same plane as the attachment surface of the metatarsal I (1); J-shaped; the articulation of the hallux is perpendicular to the attachment surface (2); the distal half of the metatarsal I is laterally deflected so that the laterodistal surface is concave (3).

233. Metatarsal II tubercle (associated with the insertion of the tendon of the m. tibialis cranialis in Aves): absent (0); present, on approximately the center of the proximodorsal surface of metatarsal II (1); present, developed on lateral surface of

metatarsal II, at contact with metatarsal III or on lateral edge of metatarsal III (2). (ordered)

234. Metatarsal II, distal plantar surface, fossa for metatarsal I: absent (0); shallow notch (1); conspicuous ovoid fossa (2). (ordered)

235. Relative position of metatarsal trochleae: trochlea III more distal than trochleae II and IV (0); trochlea III at same level as trochlea IV, both more distal than trochlea II (1); trochlea III at same level as trochleae II and IV (2); distal extent of trochlea III intermediate to trochlea IV and II where trochlea IV projects furthest distally (3).

236. Metatarsal II, distal extent of metatarsal II relative to metatarsal IV: approximately equal in distal extent (0); metatarsal II shorter than metatarsal IV but reaching distally farther than base of metatarsal IV trochlea (1); metatarsal II shorter than metatarsal IV, reaching distally only as far as base of metatarsal IV trochlea (2).

237. Distal tarsometatarsus, trochlea in distal view: aligned in a single plane (0); metatarsal II slightly displaced plantarly with respect to III and IV (1); metatarsal II strongly displaced plantarly in respect to III and IV, such that there is little or no overlap in medial view (2).

238. Trochlea of metatarsal II broader than the trochlea of metatarsal III: absent (0); present (1).

239. Metatarsal III, trochlea in plantar view, proximal extent of lateral and medial edges of trochlea: trochlear edges approximately equal in proximal extent (0); medial edge extends farther (1).

240. Distal end of metatarsal II strongly curved medially: absent (0); present (1).

241. Digit IV phalanges in distal view, medial trochlear rim enlarged with respect to lateral trochlear rim: absent (0); present (1); greatly enlarged with the lateral trochlea reduced to a rounded peg (2).

242. Completely reversed hallux (arch of ungual phalanx of digit I opposing the arch of the unguals of digits II-IV): absent (0); present (1).

243. Size of claw of hallux relative to other pedal claws: shorter, weaker, and smaller (0); similar in size (1); longer, more robust, and larger (2).

244. Alula: absent (0); present (1).

245. Fan-shaped feathered tail composed of more than two elongate rectrices: absent (0); present (1).

246. Sternum, outermost trabecula: mainly parallel to the long axis of the sternum (0); clearly directed laterally (1).

247. Distal end of furcula relative to sternal margin of coracoid: proximal to or level with the sternal margin of the coracoid (0); well beyond the sternal end of the coracoid (1). When coracoid and furcula are not remained in natural position, then their proximodistal lengths are compared.

248. Scapula and coracoid: fused (0); unfused (1).

249. Scapula, acromion process length relative to the length of the humeral articular facet: less than half (0); nearly equivalent (1); longer but less than two times (2); more than two times longer (3); (ordered)

250. Alular digit (I), phalanx 1: longer than the phalanx 1 of digit II (0); shorter than or

equivalent to the phalanx 1 of digit II (1).

251. Coracoid, width of the sternal end relative to the length along the shaft: approximately half or greater (0); between half to 1/3 (1); less than 1/3 (2).

252. Coracoid, sternal margin: convex (0); nearly straight (1); concave (2);

253. Humerus, deltopectoral crest, distal end recedes abruptly with the humeral shaft: present (0); absent (1)

254. Tibia/tarsal-formed condyles, intercondylar groove: mediolaterally broad, approximately 1/3 with of anterior surface (0); less than 1/3 width of anterior surface (1).

255. Metatarsal IV, distal extension of the metatarsal IV relative to the metatarsal III: shorter and proximal to the proximal margin of the trochleae III (0); shorter but reaching distally further than the proximal margin of the trochleae III (1); approximately equal or surpassing the trochleae III (2)

256. Reduced claw in digit IV: absent (0); present (1).

257. The length ratio between tibiotarsus and tarsometatarsus: 2 or larger (0); between 2 and 1.6 (1); smaller than 1.6 (2). When distal tarsals are not fused with metatarsals, metatarsal III length is used.

258. Pedal digit, penultimate phalanx, longer than preceding phalanges in each digit: absent (0); present (1).

259. Proximal phalanx of hallux, the longest non-ungual phalanx: absent (0); present (1).

260. Phalanx in digit IV: not as follows (0), the second and the third phalanges reduced

and significantly shorter than the fourth phalanx (1), as before but with the proximal phalanx reduced to be nearly equal in length with the second and third phalanx (2).

261. Pedal digit III claw, length relative to the tarsometatarsus: less than 20% the length of tarsometatarsus (0); 20% – 40% (1); extremely elongated and measuring more than 40% the length of tarsometatarsus (2); When metatarsals are not fused with distal tarsals, the length of metatarsal III is used. The length of ungual represents the linear distance between proximal end (position equivalent to flexor process) and tip of the sheath. If the sheath is not preserved or disarticulated with bony ungual, it should be scored as “?”. (ordered)

262. Post-dentary mandible: not as follows (0); dorsal margin concave and ventral margin convex (1); sigmoid (2).

263. Premaxilla, preorbital portion occupying 60% or more the skull length: absent (0); present (1).

264. Pedal digit II: not as follows (0); much robust than the other digits (1)

265. Alular digit (I): long, exceeding the distal end of the major metacarpal (0); subequal (1); short, not surpassing this metacarpal (2). (ordered)

266. Tarsometatarsus, length compared to femur length: 0.6 or fewer (0); 0.8-0.6 (1); 1-0.8 (2);1 or greater (3).

267. Quadratojugal, shape: without horizontal process posterior to ascending process (reversed “L” shape) (0); with horizontal posterior process (i.e., inverted ‘T’ or ‘Y’ shape) (1).

268. Jugal and quadratojugal, fusion: absent (0); present, the two bones are not

distinguishable from one another (1). (ordered)

269. Caudal vertebrae, change in morphology of free caudals along the tail: present, with distinct transition point from shorter centra with long transverse processes proximally to longer centra with small or no transverse processes distally (0); absent, vertebrae homogeneous in shape, without transition point (1).

270. Caudal vertebrae, location of transition point along the tail: begins distal to the 10th caudal vertebra (0); between the 7th and 10th caudal vertebra (1); proximal to the 7th caudal vertebra (2) (ordered).

271. Coracoid and scapula, angle between bones at glenoid: greater than or equivalent to 90° (0); smaller than 90° (1).

272. Ischium, distal end: continuous with the proximal shaft, rendering the cranial. margin of the ischium straight or weakly convex (0); directed cranioventrally toward the pubis, rendering the cranial margin concave (1).

273. Ischium, caudal margin with a dorsal process: located proximal to or close to the midpoint of the caudal margin (0); distal to the midpoint of the caudal margin (1).

274. Sternum, cranial margin with a pair of craniolateral processes: absent (0); present (1).

275. Premaxilla corpus: dorsoventral height greater than or equal to craniocaudal length (0); dorsoventral height smaller than craniocaudal length (1).

276. Furcula, omal tip: blunt or expanded (0); tapered (1).

277 Alular metacarpal, cranial margin: expanded cranioproximally and craniodistally, and constricted just before flare of articulation with digit (0); broadly convex (1).

278. Alular digit, phalanx 1: straight (0); bowed (1).

279. Major digit, phalanx 2: straight (0); bowed (1).

280. Minor digit: with four phalanges (0); less than four phalanges (1).

Supplementary Note 4

Character coding:

Dromaeosauridae

000000000000000000000000000000000000??00000000000000000000000000000

00001????1?00?0000000000?00000000000020?[01]??001000000000001000000000

0000010000000??000?000000000000000[01]0000000[01]000000000000010000[01]

00?00000000010000000000000000000[01]00000000000?0000000000[01]000001

000100100000000000

Archaeopteryx

00000000000000?0010010?00?000??0?000???0000000?1000?100000??00000?0002

0001????0?00?0000000000?0000?0?0000000????????????000?00011000000000000?

??00000000??0010000001000000000000000200000001?0001?010002?10?00000?000

1?0??0??00000000000000?0?00?000100?0100000?00100010000100002000?100000

Jeholornis

[01]?23111????????????0?????????????00?????000?00?1??0????00??10000????0100

00??????01001?000001??1201?0??1??00[12]?????????????0000000000?0[01]00??0

1011110?00000121100100001000200001001300000000011?101?010001????0?1?0?0

??0[01]0?10??1100000000??00?0200?0010?00?1?00100101000?0000000021000000

100

Chongmingia ??00???

?????[01]???????00???????0??0????000???000??????0000?????????????001??00010

??00?0??01?0?10????000?21??00000010001001????[01]30??????????????[12]??000

?111?0????1?001??00??110000000000002??0100?001????00?1?[01]111?10001??001??

??0????0010?

Sapeornis

10100102?0??1???0010?1?0?????????000???000000?01???0100[01]01??10010?002

4000000?01100?0110000000?0201000010002?????????????0000000011000000001

00111????00012110000110 0?0020010101030000??0?11010111100010101202 00?10

00?0?111?11000000000000000000?0011???0100000000101010000000 1?000?000101

Confuciusornis_sanctus

10231112?0?????00010110000120???11?112101001100?101?101[01]011120010000

24000?00001101?0?0?0000?0?020?0?00000002[12]10??01121000000000000210?0000

0101000010021000121000001100000000000210020000000000101011110003101 1202

01?1?0010000?1110000100010210020000010 00?00?00000100001100000001?00001

01010

Changchengornis_hengdaoziensis

1?23111??0?????????1??????0???????1?11??0?0??100???[12]?????????2??1???024??

0?0000??01?0?0?000????020?0??0?000021?0??01??10?0?0????02??????????????01

?????0????0?0000?0??000000021002???0???????????[12]???03???????????0???????

??110000?0?0?02??0100?0011?0?00?0?00?001001100001??1?00???01000

Eoconfuciusornis_zhengi

[12]?231?12?0??????0????????????????1?01??000??100????0?0?00???0??1???0241

???0??0??00?0???000000?020????0000?02[12]???????????0?00000?100?0???000???

?01?????0??01??00001?0?0000000210020??0??0?????????1000[12]??????????0????

??10????0?000000002?00200?001000???0??000101001000001??1?????101010

Confuciusornis_dui ??23111????????????????0?????????1???????0??1?????????????

?????[012]????14?10?0??0??0??????????????????????????2[12]?0??0???10?0?0??00

021?????????????01?????0???1??0[01]00?00?000??0021002?????????????????????

???[02]?????????1???????10????0?00????0[12]00?0010?0????0??0?1000011?00[01]

0001?0???1??010

Boluochia ?1[12][02]??

??????????????2????????1?????????????????????????????[12][12]?[12]??????20????

???110101???02??

????????0????????????0[01]?100100002??3200?0011??1????????2?20??????????1???

??1?????

Concornis ??01?

?11????????0?01???????111110?02?11?00?1?????111221222111?20?00111??0[01]0

211??????1????1112?????????????0??0???30010[01]0122??????????11?1?[12]??00?0

?????????1111???????1110?0010??0[01]??0000?001???01131110?0?2??1???022??1?

1??1?0??01

Elsornis ??[01]??10?0

?1?10???????????????????111110002121?000001010111[23]22010?11??[12]0?00110

000000[12]0[12]0000111??0100[12]1?1??31?????0?[01]?01?????????????????????

??1111?121????????????

?????1??0?0????

Eoalulavis ???00111200?

?2?0???????????????0??11111010211100000010?111?312?0?0010?40?00111100102

11001001111111?21?1?????????0??1?01?001????2???0?????1??????????????1?1??

?????????????????????????????????????1??1121111????????????1?????1??0?0?101

Cathayornis

[12]0[12]0??0??????????????????????????000????00?????1??1??1??0???11?2????24

0?0?0111???1111?0??20???000001?0111?322122311??20?0011?1?010211[01]01011

111011????110?31100010?[01]00013001010122000000??11??????100????12????1??

??[01]?????1?1?????????????????????????0?1111111????????0?2???1?10?01?1101

Eocathayornis ???0???2???????????????0???0?????000????00?????1????????00?

?????????????????????11?????02????000000??0????22121311??20??01??1?000???

[01]????11???10??????0?31??0010??0001200100011?????????????????????????????

???????????????????????????????????????0?111111???????????2?????1??01?1101

Eoenantiornis

[01]010??02???????????????0?????????00????????????1???????????????[012]????2

4020?????1?0111110101?11000?001???11122[123]?2??110?2000?1??1?000????????

?????11?????10?31??0000?00001200101012???0???????????[12]??001????1??????

???????????11??0?10??????0??1?0?1110011[23]1011?[01]?1?0??20?11??1?10?0001

101

Gobipteryx

10[12]3101200021?10????????0???0?1??1?[01]0???10???10??????1?????????[012]1

0??[12]401?????????1111?010?1???0??0010??111[23]?????????????0??????0??????

0????????11???111?????????0??0?01?00??????20??????????????10?03???????????

10[01]10?011?110010?100??11?0??1?1??????????2??1????????0???????1???1?????

Longipteryx ?122111??????????????????????????000????00??01?1??1011????

?????2????24??0?01?11?01?1110100????000?000?111132[23]?21111??20??0110??0

002??[01]?0?011?1?11??????0?01100000100?012001000113000????1111011[12]11

001?0?[12]1??0????0????0?1?1?0?1000000001?32000001???1?1211?1?2?2????01?1

1??1?1000101101

Longirostravis

[01]022??1??????????????????????????00?????00??01?1??1?????0?????01????24??

0?01110?0111????00?2??000000??0111[123]2[123]?224110?20??01??1??00???[01]?

????????10??????0?31??10?0?[01]01012001??0122??0?????1??10?1?11????0?1????

??1?00?????1?11[01]?10010??0?2?0100?00????0?1??111?1?1????01??1??1?1??010?

???

Neuquenornis ??0??

?11????????????????????1?1????021111000????1?1111230[12]?1?1?????0????11????

?1?01?01????11?1??11??31??10?0?[01]0001???????????????????????????????????111

0????????1????????10??0?1??1?2?00?011001[12]????1??2[12]0???1?0????0???????1?

???01???

Pengornis

00[01]01101?0??????01?????0?1???????000???0?0?????11?2??1??0?1?10?1????240
?0[12]??????01?11???00?????0??02???11?[23]?????????????001?0100101??[01]?10
0?1???11??????1[02][23]111???0?01001?0010?0?[12]3??????????????????02?01??
?????1??[01]?01[12]11?0100100100101200201?00?????011?100?[01]011?02101?0?
?1?1???10??01

Eopengornis

0000110[012]?0??????????????0?????????00000?000?????1??10?0??00?????????24?
?0?1?????01211101000???000?02?00111321?110?1??10?0010010000??10?0001????
10??????0??1[01]?00?0?010012001010103?????????????????000???????0????100?
?????0???0001????1??0101?0012?000111101?1011102?0101??1?1??0101101

Protopteryx

[12]?[12][02]1??[012]?????????????????????????00??????00????1???????10?????01
????14????0??1???1111?0?11?[12]??200?00??0111322111011?[12]10???1????010??
???????????11?????????1??000??[01]0001100000010???00????11?????????0[12]???
?1?????0???????????00??0010??00??0[01]01?001110011?0100?[01]101101?0000??1
?1000101101

Rapaxavis

[01]022??1[012]??????????????????????????000???0?0???1?1?????1??01??11?1???02
4??0?0?110?01????0?00?[12]??0??001???11?322122411??20??0100??0000??[01]0??
?1111?10???1??0??11?00?010000130011?01230000?0??111101?111?0100????????0
?0[01]?0?11??100?10000??002?0100?0011??0?121011?10110210102 1??1?100?1011
01

Shanweiniao

[01]?[12][23]??1????????????????????????????0????00??01??????1??????????????

24?????1?1??01?11???000[12]??000000??011?22[123]?211?[01]??20???1?????00???

?????11???11????1?????????????????20?11???22?????????1??????1????0??????0??1

??????????110??0000????1?[01][01]?0?0?1[01]?10?12?111?1011?21010[01]???1?1?

?010??01

Vescornis ?0?????2??????????????????????????0000???00[12]001?1??21?1?

?0???1??2????2???????00??01?1??0?02?[12]??000001??011?322120111??20?00110

??0101?1[01]0???1111111??????1[02]011010?00[01]0?0130010[01]01220??0??????

????????????????????0??[01]????????00??0010??011?[01]201?1011??11121[01]11?

[01]1110?00?021??1?1??0?01101

Qiliania ???

??

???10????00?10101?2?0003?0??11200?00?1

10000?11100?001001?11?[01]?20?0?1[01]??????????1?120021??1?2?????00???????

Dunhuangia ???

?????????????????????1111?0?02120?02??0??1?11?[23]2[123]?2121???200?0?10??

000?????0001?????1??????0?3110000010010120010001 2????????????????????????

??0?1?1021???????????2?????1??0?01

101

Piscivorenantiornis

1??[02]???????????????????0?0?0?0???000????00??01011110111?001?[01]1?[12]11

?02?000????01?0111110102011002000 1?01111222121310??30?001??100001010000

01111110??0?110?2110000010010120010???231000?00?0?010111100?20011?0????

1??0?01001?1100100100102?0020110?1???0112102111?1????0??21??1?1001?0110?

Linyiornis

10101?02?0?????????????0?????????000???0?0??01?1??10?1?2?1?0110101??24??0

20?0???0111110101[12]1?0020001?00111[23]2[123]?[12]??1??????00100??0102110

0???11101111001 1?0121000000100101 20010???22000??0010?0101?1100??0012?0?

???1?0?10?001?110010010??0?0?0201?0011??1112102111110 02100?20??1?100000

?101

Sulcavis

[01]010??0????????????????0?????????000???0?0?????1?????11?????11?[12]????24

?1010110??01????0?010[12]??0[01]0001?0?1113[12][123]????????????01????010??

???????1???11??[01]???0?[02]1??0[01][01]0?00?0120010101120?00?[01]?????????[

12]10?????????????0?00?0?01??110010010??0[123]??0101?0011??11211[12]01111

0012?0110??1?1????01101

Bohaiornis ?0[12]01102?0?????????????0????????1000???000??0011????1?

120?1?110[0123]????24000?01101?0111110?01020?0[01]0101?0?11122[123]?21311

??30?001??100001?10?0?011??11?????10?0110000010010120010101230000??????

01011[12]??000?0?1????0?0??0?0??10?1100100100?01?[01]0101?0?11??101210211

110001210110??1?1000001101

Longusunguis ?0[12]01?02?0???????????????????????000???000??01?1??[12]?

1??20??1110[123]????24?00?0110??01111101020???010001?0111?32[123]????1???[

23]00001??10000??10?00011???11?????10?01??0000100101?00101012200????????

01011[12]??000????1?100?0?00?00010?100?1???000?1??010100?1????112102011?

1002210110?01?1000001101

Shenqiornis

[01]0101102????????0??????0?1???????000???000???1?1???11?0?01??11?????????

0???????01?11?0?01?[12]??010101??011?[23]2[123]?[12][12]3?1??20??01101?000?

????????1???11????????01??00101001012001010113000??????01?111???01??????

????0?????????????0?1011???0???[01]?0?0?1???[12]111211[12]01??2?0?21011??01?

1000001101

Zhouornis

[01]0101?01?0???????????????????0??1000???000??0??11?[12]?1??20???110?????2

4??0??1?0??01111010101??020101?0111132[123]?2?3110??0?000??10000??10?00

011???11?????10?31??0000100101200101012200000??????????[12]??00000??1?1?0

?1?00?0?01??110010010??01??0101?0?11??11121?2011110012?0110??1?1??000110

1

Parabohaiornis

[01]0201102?0??????????????????????000???000??0??1???01??200??110[12]01?12

4??0?01101101?1110?01?11?010101?0111132[123]?20311?[345]30?001??10000101

010001110111?????10?01??000010010120010101230000??0?0101011[12]1100000?

?????0?0?00?00010?100010010??01200101?0?11??111210211100022?0110??1?10

00001101

Fortunguavis ??????0????????????????????????????????00???0????0?110200?

?1?000??02[34]??0??1110?0111110100111002 0????0111122??2??1???300000??1000

0??1??0001?1??11?????1122110000010010120010 00?120?????????0101111001 2?0?

???????1???10???1?11001001 0000?0?02?100011???11?10?1?11100111?011??1?1??0

??1111

Pterygornis

00201102?0??????????????0?0?0?????000???0000001?111[12]?11??0111110211?12?

000??1101??1????0102111000000 1?0?11132[123]1212100?20?00100100001110000

011001110000110?31100000100101200???01221?0?10011??10??1100?10011?0????

1?001????1?1100100110101?0010110?11??011?1021?1?100??000210?1?10?11011?

1

Cruralispennia

[01]0?0??02?????????????????0?????????000????0??????1????????0?112????????2??00

????0[01]??1?1??01011???00?001????????2[23]12201???10000110??0001??????????

??10??0?????[23]1??00???0??0120??0???2200????????01011110003???????????1???

???????1100?00?00??????????????????1?100221???2???1????2??1?1?101??10?

Monoenantiornis ?0?0??1??????????????????0???0????000???00??????1?????????0?

??1??1112?2???????1?0???1??110??1??0?0000010??11??231200000????10100??0001

010????1100011?????00?????0??0100101200000??12???????????????????0000??????

??????1?0111??1?0?10?1???0100?2?0?0?11??1?1?101012000012??110??1?10?0101

101

Archaeorhynchus

1013111[12]???????????????????1?????????1?00??00100??1???01?0??0??1???1000??[34

]0111????1101011?0010?20?[01]20000?0121002[23]122211?[12]20??1000??010???

?0???0110?100001100?31??00011000002101010103000??0?101100?[12]10001?0?

???1???1?0[12]00????211[01]010100??0?0?0100?00????001010110000?01[01]002

0?01?10?0110101

Schizooura

0123111[01]??????????????????????11?0????10??01??0?[12]1?00?00?1??0[345]?

1??[12][34]?[12]0[12]?000??01011?0?1001??[01]2?00????11012[23]0[12]?011??1??

1?000??0[12]0010[01]0???01?0?10?????00?31??0000?001?0[23]001010103?0??1[0

1]1???0101?1?0103?????[12]?0?????[12]???????111010?00001[01]2?011000010?1?

01?1110?100000010021?01?1??01?0101

Bellulornis ???1?

00?[234]????2?01?11000???1011?0?10?10?020????0?11?12[23]0[12]2211?????1100

00?0100100????01???1000???00?310000000000102101000103l???10?1??010?1210

0???0102?????1?02?0???1?112010120???02?0200?00?0??001?0011?1010?00??021?

?1?10?1??1101

Vorona ??

???

???101120211?0001

111011111[12]000100011?21000000?????????????11??????????1??????????????

Jianchangornis ????????????????????????????????00????1?0?????1??1?????010

1200[234]0???????????????010111001002 0?0200?0?0121?02[23]031001??[01]0?01

0000001001000000001?0111?????0???1??01?00000002101010103??01?[01]????????

?[12]?000?????2??00???0??0?10??1120?0000??????0100?00???01010001110000?0?

10?01????1??1?1100?

Songlingornis ???[01]?????????????????????????????000?????0?????1?????????

???2????????????????????10?????101???2????0???21?02301231[12]??51??????????

???0?????????

??????????????????????????????????????1?10?01??????????????????1????1????

Longicrusavis

10[12]01?0??????????????????????????00???101??01????11?11?0???20??????????

?????????1011100?0????[12]21100?01210?23?120110?10?110?01?11012021100010

001100???0??31110??00010102100010101???????11??1???[12]??0?????????01?100

22[01]121?1112011100??10100100?0011??0010101000110000???02????1??011?101

Apsaravis ??????????????????????????1[01]?????1?1????1????????021?1?2

01??00?4??0?24?10????????101110000111121102001????[12]30???11?????110010

0010111000011111110121101?311001110110???1????????100011111?111012??113

101[12]?1?0??1???20121??11[12]?111001?1?200100?00??????1??12110??0?????0?1

????1?????0??1

Hongshanornis ?020?10??????????1????????????????00???101??0?????????????

??1????????24???????01?0101110010????[01]211?0?0121?12??2201[12]?210?110???

??10???2????0????11????????31??00?0??00?02101010101??????????0??01[12]????

3????????????0?[12]???????1120?0?00?0?0??0200?001111001010210011000010012?

11?1??0110101

Archaeornithura ???

???[23]???0[12]4??1110001?0101110010110?221100?0?2101230[12]?111???????0??
10010??02????0100????????0??[23]1??0[12]?0?000?02100010101?0????????01001[
12]???03????2??0??1?0????2?0?1110?1?01???[01]??0100?001111?0101011?011000
01???1??1?10?0??0101

Parahongshanornis ???1?1?????????
????????????????????1????0?10????[01]211???01210123022011??10??10??1?010?
?0?????01???11????????31??01?0?00010210001010 1?0?????????10??[12]??003????
???0????00???????1???11?0????1??0100?0011??001?1011?0110000????2????10?0?
10101

Tianyuornis
1020??0?????????????????????????000????01??01?1????????????????????4??????
??11?10???0?10????22??????1210123?22111??10?1100???010??0?????01?0?11????
????[23]1??0??0?00010210001010 1??????????????????????????????1??[12]??12??
?111011?01???[01]??01?0?0?11??001?1011?0110000????2??1?1??011?101

Yanornis
10[12]11102?1????????1?????????????1000???1?00?01?1??0?????01??2003??0?2??
????000??01011100100?0?220001?0121002301121[12]??51011001??010110[01]???
?0100011?0?11?0?31110000000010210101012310100?11?10100?11?003??1???????
1?02?0??0??111011?00[01]1112?0110?0011??0?10100100000000000011?11?11?1110
101

Patagopteryx ????????????????1?11110001111????????????0??000?112110110
01100031101??120????????1011100000?0?1210010??????2[12]????10?????1001???

1000?0?00000100?000000?0?????????0??1?1?20?00???20?0001000001110121?013?

0??20????1001?0?01111120101001110000?00?0001????10??110100000?0?0???????

0????????

Yixianornis

1121???????????????????0???1?1???0?0???110?????1??1?1??2011?1003??0?24????

1000[12]0010111001001 0?22000100121 0?2301121[12]0?51??10??11??0?0?0?11001

?0?100021?0??31110101001010210101010 2100?1??00?01?111??003??1?2?201?100

2???????11[12]01?0001110?0010??0011?110101011?0000001?0011?11??1?1?10101

Piscivoraavis ???????????????????1????111?[01]????????????????????1211??10

111100[34]??0?24?20??0001001011?0??0????[23]20????0121002301121[12]??51?1

10??1?1?0??0??????1?0?11?????00?31??000110001021010101121000?0????01011[

12]10003?01121?01?10022????1?112011100[01]110?0011000010110 01?01???00000

00??001?11?11?0?10101

Iteravis

00231102?0???????????????0???????000???1?0??01?1??[12]1????01?12?0[123]???0

24?20110001101011?0?10?10?220????0121002[23]01111[01]??51?11000??01?1?02

????01??11?????01131210101000110210101 0122100?11111?01011110000?010212

0??100[12]?1?00??11211110???100?0110?0011??101?101?0101000000022?11?11?0

110101

Gansus ???121?1?201

111004001024?20210000?11011100100 20?320???001210023021 0100?10?11001??0

10130?1???0100111001 11?0?312?01110001?131010001221?001111010101 1210003

001[12]?1201?100[12]2?0?0?2112011100101 02?020000011??001?10110?010000??0

23?11?11?1?10101

Ichthyornis

20231?03100?1??1111111011111 11?01001???100?1011111[01]11012011120?4?0102

401021000??1101111010010?2210110012?002300??1212?0111001100101 20210110

1000110021101?3121012101001031111???3100010110?1101121011310122 1201?1

00221110?2112011202101?2102000001?????10101001?1????0?????11?10??100101

Hesperornis

11231101?0021??1111111001011111 01010???10001010 1?021001211112004010?130

20210001010?01?00?0010?020???000200?21?0??0011?00??????0?????1000000????

????????????????????????????????1000121101110012101131021222011 10122111

1121120112?21013223220102??????1??01?020?0?0?0???3?11?00??1?????

Parahesperornsi

1123??0???0?1??1111111?010111????01????1???????1?02100121111200?????1?????

10001??0?01?0??0010?0????????????2???????1????????????????????????????????

???10212220111012 21111???1201

?2?2101?223220002?????????????20?0?0?????3????1?????????

Enaliornis ?????????????????????????11??????????????????????21?0??1???

2???

???????????????????????????????????????1??????1???????2?????10122120111012

21111???1201???1101???0120000????????????02?????????????????????????

Baptornis_advenus

1??3??????????????11???0??111???????0??1?0?10101?021001?11112004010?13020

?100011?0001?0010010?0?????0??????2[12]????0112??0???????????1000000????0

0000100????????????????????????010001211011100121011310112120111012211 1

11211201?2?1101322112000 1????????????02??????????3????00????????

Baptornis_varneri ???021?01?1??1

???

?????????????????????????????????????10????1???1100?210113?????2201?1012

211111211201?2?1101?221220001?????????????2???????????????????????

Vegavis ??2????????

????[56]?????????????????10111001001?0[01]20100?01????????????????1100111?

??0??????????????????012?1??02211001003 1111??12301???1?????01??2??????011

21201?1002?2?1????12011???3?1????1???0???????101011???????????23????1?????

00?1

Anas

21230113101111111111111111112112111?102100001011?10211012111100060120240

20210002011011110100 20?22010000121002311100113401110011110003011111010

000100111012312013211111103 1011??123110111110101111210113101121201 1100

2220111211201120130132102200001011101011210101000100023?11?10?1100???

Gallus

11231113111211111111111111112112111?102000001011?10211012111100160120240

20210002011211110100 20?22110000121012311010112201110011110003011111110

001100111011312013210210113 1011??121110112110110111210113101121201 1100

2220111211201120130132201201001011101011210101000100022?11?10?1100???

Yangavis

[12]0231112????????0????????????????1?0???01001100???10?1?[01]0??10?01???0?

?0?0?????110??010??00????020?0??0000002[12]?0??0???10?000000?02100000???0

1???01?00?00??210?00001000000000002100300???????????111??003?0?1??????1??

0?0000?0?10000100??0?2?0100?0?11???00?1??001000000100000??1?0000101010

Shangyang

20[12]0??0????????????????????????????????00?????1????1??????????2??0?24?00

20110??01111101020???0[01]0001?0011122[123]?2?310???00001??10000??00?000

11???11??0?11??21??000010000120010001120000??0?0101011[12]1010000????100

?1???10?00??1100100110002?0000110011??01111111?1010021?0021??1??0011001

01

Jinguofortis ???0??0??????????????????????????000???0?00001?1??[12]0?0?2

??010?01????24??020??0110??0??0????????00????01000021????00????0001001?01

00?00?0??0100111??0?000?21??000110100010010101130000??0?0?0101111000[01

]?01??01?0???00????????100?000?000??11200?0?12?0?00?1??0?211000??0000??1?

0000?10101

Mirusavis ??00?10????

??????????????????1101????0?02?1100??001?0?11122[123]?21310??30?00110??00

012000???111?111012010??21??0000100101200100??2??????????????????????????

?0??????11?????1???????????????????????????001?1121???????????2????????1?11?

01

Mengciusornis ??20?????????????????????????????1??0???1?0??0101??0?????0?

112??311????????????11010111001002 0?020102?011101?????????????1?000??0[12

]00?001???0101?00??01100?31110000100110200101011 31???11?1???????[12]1000

3?0?1??????1??2?????1?1110?0100??1?0?0200?0?10???01?1110?0010000?0021????

1???110101

Similiyanornis

00[12]1??0?????????????????????????1000???100??00?1??0010??01?????3????2???

1??0001??1?11?0010?10?22?001???2100230???1??????1100???010100?0???01?0?1

1?0?110??311?0000?00010210101011 3??????????0100?[12]??0030?????????1?02?0

?????11101?101??11??01?0?0?1?????1010?1?001000000011?????1?11?0101

Abitusavis

0??1?????????????????????1?1?????000?????00001?11?0010??01112?0211??2???1?

?0001101?1110010?10?220001???21002301?01???51?1100???010??010???0100011

???1100?311100000000102101010123???????????????[12]??0030?????????1??[12]?

??????111011100??11200110?0?????00101011?00100?00???1???????0?10101

Chiappeavis

012?????0?????0???1??????????????0000??0?0?????1???1????01??1?02????24?

[12]0[12]1??0???1111010001??000?02?0?11122[12]?12011??10???000100000?????

??11?1111?????01[02]211????0?00?01?00?0??1?30000????0101???1??100??????0??

??1[01]????1??0??????1????1???????0011?1?0111?0?10?11???2000?[01]??1?10?000

1??1

Parapengornis

00[01]0??0???????????1????0???00????000???0?0??0??1??[12]?1???01??1?????

[12]?24?10?10?01?0111??0?000?0?200?02??111?3[12][123]?1?01?0?100001?0?000

0?????1??0????111?????1[02]?1??0010?01001200100001300?0??????000?1?10110?

?????0?????000?110??0?0000?10???1??01?????11??011110010?110112?0020??1?10

?0001101

Eogranivora

?023??1??????????????????????????1?10???????????????????????????????24?[1

2]1110?0???1011?0?10????[01]2??????121??23022211???0???0????010????????01?

??1??????01??1??01?00000?02101010113?000????11??????1??13??????????1?0????

???211[01]010?0???????0000?00???1??1?11[12]1?1?00?01?0020??1????0100001

Gretcheniao

???[02]??0????????????????0?????????000???000??01?1??[12]??1010??1110???

??241?0?1??01?01?1??0?02?11000?001???111323121[12]1???20?00110??00011000

???11000111???110?211000?0100101100100012200?0??0?1101011[12]10001?0??10

1?0?0?0[01]??0011?110010010??011?0000?0?11??1112112111?1?0??00121??1?100

0?01101

Xinghaiornis[01]123011[01]?1????????1??????????????1??????10??0??????????????

????[234]????[12]4??0?1000???1011100100???2201?0??111?[23]2[23]?[12]22111??

??010??00020?3?[0

1]?????????11??2?10?????????1?00??0?1010?01131000??0??1?101?21????????2??0

??1?1?1????0?11[12]01???001?0??01?0??010??0?10??11?0010000?10?1??1??10010

??01

Dingavis

[01]023??1??????????????????????????1?10???000???????2?????????????????24???

??000??0?0????????????20?00??1????2[23]?[12]???????0?010??00000?????1??????

?1?????????21??02?1?00000210101010102?0??????11??11[12]??010?02[01]2?[12]0?

?1??[12][12]??????112011???00102?02[12]0?0011????1?1??1?002000001023??1??1

0?1?0101

IVPP V12707

0011010101?101?1?02411??0???

??00?10?111?101???0???001

110??00?00??????1?1???1??1???????0??0000?????????????11???1?000??001??00?0

?????

Supplementary Table S1. Select measurements of IVPP V12707.

Element	Length (mm)
Skull	21.21*
Pygostyle	10.05
Femur	14.32
Tibia	16.69
Metatarsal II	9.44
Metatarsal III	9.87*
Metatarsal IV	9.08*
Pedal digit II-1, 2	

Pedal digit III-1, 2, 3, 4	2.82, 2.53, 2.96, 3.78
Pedal digit III-1, 2, 3, 4, 5	1.64, 1.47, 1.51, 1.69, 2.89

Supplementary references

1. O'Connor, J. & Chiappe, L. M. A revision of enantiornithine (Aves: Ornithothoraces) skull morphology. *J. Syst. Palaeontol.* **9**, 135–157 (2011).
2. Wang, M. & Zhou, Z. Anatomy of a new specimen of *Piscivorenantiornis inusitatus* (Aves: Enantiornithes) from the Lower Cretaceous Jehol Biota. *J. Vertebr. Paleontol.* **40**, e1783278 (2020).
3. Baumel, J. J. & Witmer, L. M. in *Handbook of Avian anatomy: Nomina Anatomica Avium* (eds Baumel J. J. et al.) 45–132 (Nuttall Ornithological Club, 1993).
4. Balanoff, A. M. & Norell, M. A. Osteology of *Khaan mckennai* (Oviraptorosauria: Theropoda). *Bull. Am. Mus. Nat. Hist.* **372**, 1–77 (2012).
5. Hu, H. et al. Evolution of the vomer and its implications for cranial kinesis in Paraves. *Proc. Natl. Acad. Sci. USA* **116**, 19571–19578 (2019).
6. Field, D. J. et al. Complete *Ichthyornis* skull illuminates mosaic assembly of the avian head. *Nature* **557**, 96–100 (2018).
7. Gussekloo, S. W. S. & Bout, R. G. Cranial kinesis in palaeognathous birds. *J. Exp. Biol.* **208**, 3409–3419 (2005).
8. Sander, W. S. G., Vosselman, M. G. & Ron, G. B. Three-dimensional kinematics of skeletal elements in avian prokinetic and rhynchokinetic skulls determined

by roentgen stereophotogrammetry. *J. Exp. Biol.* **204**, 1735–1744 (2001).

9. Dawson, M. M., Metzger, K. A., Baier, D. B. & Brainerd, E. L. Kinematics of the quadrate bone during feeding in mallard ducks. *J. Exp. Biol.* **214**, 2036–2046 (2011).

10. Claes, R. et al. The effect of craniokinesis on the middle ear of domestic chickens (*Gallus gallus domesticus*). *J. Anat.* **230**, 414–423 (2017).

11. Holliday, C. M. & Witmer, L. M. Cranial kinesis in dinosaurs: intracranial joints, protractor muscles, and their significance for cranial evolution and function in diapsids. *J. Vertebr. Paleontol.* **28**, 1073–1088 (2008).

12. Wang, M., Lloyd, G. T., Zhang, C. & Zhou, Z. The patterns and modes of the evolution of disparity in Mesozoic birds. *Proc. R. Soc.* **288**, 20203105 (2021).
