# Supplementary material for: Cretaceous bird with dinosaur skull sheds light on avian cranial evolution
Source: Nat Commun. 2021 Jun 23;12:3890. doi: 10.1038/s41467-021-24147-z (PMC8222284; doi:10.1038/s41467-021-24147-z)
Supplement: Supplementary file 2 — Reporting Summary [file 41467_2021_24147_MOESM2_ESM.pdf]

nature research

Corresponding author(s): MIN WANG

Last updated by author(s): Jun 2, 2021

Reporting Summary

Nature Research wishes to improve the reproducibility of the work that we publish. This form provides structure for consistency and transparency in reporting. For further information on Nature Research policies, see our Editorial Policies and the Editorial Policy Checklist.

Statistics

For all statistical analyses, confirm that the following items are present in the figure legend, table legend, main text, or Methods section.

n/a	Confirmed	
☒	☐	The exact sample size (n) for each experimental group/condition, given as a discrete number and unit of measurement
☒	☐	A statement on whether measurements were taken from distinct samples or whether the same sample was measured repeatedly
☒	☐	The statistical test(s) used AND whether they are one- or two-sided<br>*Only common tests should be described solely by name; describe more complex techniques in the Methods section.*
☒	☐	A description of all covariates tested
☒	☐	A description of any assumptions or corrections, such as tests of normality and adjustment for multiple comparisons
☒	☐	A full description of the statistical parameters including central tendency (e.g. means) or other basic estimates (e.g. regression coefficient) AND variation (e.g. standard deviation) or associated estimates of uncertainty (e.g. confidence intervals)
☒	☐	For null hypothesis testing, the test statistic (e.g. F, t, r) with confidence intervals, effect sizes, degrees of freedom and P value noted<br>*Give P values as exact values whenever suitable.*
☒	☐	For Bayesian analysis, information on the choice of priors and Markov chain Monte Carlo settings
☒	☐	For hierarchical and complex designs, identification of the appropriate level for tests and full reporting of outcomes
☒	☐	Estimates of effect sizes (e.g. Cohen's d, Pearson's r), indicating how they were calculated

Our web collection on statistics for biologists contains articles on many of the points above.

Software and code

Policy information about availability of computer code

Data collection	No software/code was used in data collection.
Data analysis	The ct scanned data were imported into Avizo (version 9.2.0) for digital segmentation, rendering, and reconstruction, which were then optimized in MeshLab (version 2012.12); phylogenetic analysis were conducted using the TNT v. 1.5 software package.

For manuscripts utilizing custom algorithms or software that are central to the research but not yet described in published literature, software must be made available to editors and reviewers. We strongly encourage code deposition in a community repository (e.g. GitHub). See the Nature Research guidelines for submitting code & software for further information.

Data

Policy information about availability of data

All manuscripts must include a data availability statement. This statement should provide the following information, where applicable:

- Accession codes, unique identifiers, or web links for publicly available datasets
- A list of figures that have associated raw data
- A description of any restrictions on data availability

The three-dimensional models (STL) are archived and available on MorphoBank (project no. 3972), or from the corresponding authors. Data matrix used in the phylogenetic analysis are provided in the Supplementary Information.

Field-specific reporting

Please select the one below that is the best fit for your research. If you are not sure, read the appropriate sections before making your selection.

☐ Life sciences ☐ Behavioural & social sciences ☒ Ecological, evolutionary & environmental sciences

For a reference copy of the document with all sections, see nature.com/documents/nr-reporting-summary-flat.pdf

Ecological, evolutionary & environmental sciences study design

All studies must disclose on these points even when the disclosure is negative.

Study description	Comparative anatomy of an Early Cretaceous bird fossil
Research sample	One fossil specimen, IVPP V12707
Sampling strategy	No sample size calculation was performed.
Data collection	The specimen was discovered in the Early Cretaceous Jiufotang Formation (120 Ma) near Yixian Country, Jinzhou City, Liaoning Province, northeastern China. The fossil bird was CT scanned using the the industrial CT scanner Phoenix v-tome-x at the Institute of Vertebrate Paleontology and Paleoanthropology (IVPP), and the Yinghua Inspection&Testing, in Shanghai during a period from January to August 2020 when the corresponding authors conceived the whole process. The ct scanned data were imported into Avizo (version 9.2.0) for digital segmentation, rendering, and reconstruction, which were then optimized in MeshLab (version 2012.12). The morphology was analyzed based on the CT data and direct observation of the specimen.
Timing and spatial scale	The fossil bird was CT scanned using the the industrial CT scanner Phoenix v-tome-x at the Institute of Vertebrate Paleontology and Paleoanthropology (IVPP), and the Yinghua Inspection&Testing, in Shanghai during a period from September 2019 to August 2020.
Data exclusions	No data was excluded from the analysis.
Reproducibility	The specimen (IVPP V12707) described in this study is archived and available on request from the Institute of Vertebrate Paleontology and Paleoanthropology (IVPP), Chinese Academy of Sciences, Beijing, China. The CT scanning results and three-dimensional models (such as STL) are archived and available on MorphoBank (project no. 3972), or on request from the IVPP, or from the corresponding authors. Data matrix used in the phylogenetic analysis are provided in the Supplementary Information.
Randomization	not applicable
Blinding	not applicable

Did the study involve field work? ☐ Yes ☒ No

Reporting for specific materials, systems and methods

We require information from authors about some types of materials, experimental systems and methods used in many studies. Here, indicate whether each material, system or method listed is relevant to your study. If you are not sure if a list item applies to your research, read the appropriate section before selecting a response.

Materials & experimental systems

n/a	Involved in the study
☒	☐ Antibodies
☒	☐ Eukaryotic cell lines
☐	☒ Palaeontology and archaeology
☒	☐ Animals and other organisms
☒	☐ Human research participants
☒	☐ Clinical data
☒	☐ Dual use research of concern

Methods

n/a	Involved in the study
☒	☐ ChIP-seq
☒	☐ Flow cytometry
☒	☐ MRI-based neuroimaging

Palaeontology and Archaeology

Specimen provenance	The specimen was discovered in the Early Cretaceous Jiufotang Formation (120 Ma) near Yixian Country, Jinzhou City, Liaoning Province, northeastern China.
Specimen deposition	The specimen (IVPP V12707) described in this study is archived and available on request from the Institute of Vertebrate Paleontology and Paleoanthropology (IVPP), Chinese Academy of Sciences, Beijing, China.
Dating methods	The horizon where IVPP V12707 was collected belongs to the the Early Cretaceous Jiufotang Formation (120 Ma) of the Jehol Group, based on previous chronological and lithostratigraphic correlation study.

☐ Tick this box to confirm that the raw and calibrated dates are available in the paper or in Supplementary Information.

Ethics oversight	No ethical approve was needed in this study.

Note that full information on the approval of the study protocol must also be provided in the manuscript.
